# Chronic Loud Noise—Biochemical and Ultrastructural Alterations in Auditory and Limbic Regions of the Rat Brain

**DOI:** 10.1155/bmri/2680036

**Published:** 2026-04-24

**Authors:** Nino Pochkhidze, Lela Chitadze, Lia Tsverava, Giorgi Lobzhanidze, Nadezhda Japaridze, Fuad Rzayev, Eldar Gasimov, Mzia G. Zhvania, Revaz Solomonia

**Affiliations:** ^1^ School of Natural Sciences and Medicine, Ilia State University, Tbilisi, Georgia, iliauni.edu.ge; ^2^ Department of Brain Ultrastructure and Nanoarchitecture, Ivane Beritashvili Center of Experimental Biomedicine, Tbilisi, Georgia, lifescience.org.ge; ^3^ Medical School, New Vision University, Tbilisi, Georgia; ^4^ Department of Histology, Embryology and Cytology, Azerbaijan Medical University, Baku, Azerbaijan, amu.edu.az

**Keywords:** limbic region, MDA-protein adducts, neuroinflammation, neurotransmission, oxidative phosphorylation, prolonged loud noise, the inferior colliculus, ultrastructural changes

## Abstract

Chronic noise is a major environmental risk factor leading to a number of pathologies in different parts of organisms, with a profound impact on health. The auditory system is the most vulnerable to the damaging effects of noise. However, sound processing is also supported by brain regions outside the classical auditory pathways, including limbic areas. Little is known about molecular and structural mechanisms of long‐term noise effects in the limbic area. The work was aimed at filling this gap. In the present study, we compare biochemical and ultrastructural alterations, elicited by 30 days of loud noise in the classical auditory and limbic regions of adult male rat brain: the central nucleus of inferior colliculus, the basolateral amygdala and the hippocampus. We have shown that 30 days of’ loud noise exposure leads to biochemical and ultrastructural alterations in these regions. Depending on the area, significant and specific changes were demonstrated for the protein components of synaptic vesicles, inflammasomes, and the mitochondrial oxidative phosphorylation system. The lipid oxidative degradation product, malondialdehyde protein adducts are significantly increased in the central nucleus of inferior colliculus. Some of these changes could be compensatory. The central nucleus of the inferior colliculus demonstrates moderate and severe ultrastructural alterations, whereas the ultrastructure of the limbic regions was more preserved. The data convincingly indicate the interrelated molecular and morphological changes after long‐term loud noise exposure in the classical auditory and limbic regions of the brain. The observed changes may lead to pathological processes that harm the normal functioning of the brain.

**Summary**

• Chronic loud noise is an environmental risk factor leading to a number of pathologies.
• 30 days’ loud noise exposure leads to biochemical and morphological alterations.
• The changes occur in classical auditory and limbic regions of the brain.
• Proteins implicated in various molecular pathways are altered.
• Various intracellular structures are changed in a pathological way.

## 1. Introduction

Chronic loud noise is a continuous part of the physical environment in urban, military and industrial areas. As an environmental risk factor, noise produces a number of pathologies at different levels of the organism, with a profound impact on health [1, 2]. According to the World Health Organization, more than 1.6 million healthy life‐years are lost each year from damaging effects of deafening noise in Western Europe [3, 4]. The auditory system and associated regions of the central nervous system (CNS) are the most vulnerable to the damaging effects of noise. Recent studies highlight the CNS effects of chronic noise via molecular pathways and changes in the brain [5, 6]. However, many questions regarding the molecular and structural mechanisms of noise effects remain unresolved. Valuable information has been obtained from the classical auditory pathways, such as the inferior colliculus, medial geniculate body or auditory cortex, which play a vital role in the sense of hearing and sound localization [7, 8]. However, the functional neuroanatomy of sound processing is complex as this process is also supported by other brain regions outside the classical auditory pathways, including limbic areas, the reticular activating system and cerebellum [9–11]. To fully understand the consequences of noise on the brain, it is important to carefully analyze the multifaced changes induced by noise in all brain areas, involved in sound processing.

In the present study, we evaluate biochemical and ultrastructural alterations produced by 30 days of loud white noise in the classical auditory region and the regions of the medial temporal limbic system in adult male Wistar rats. We focus on the central nucleus of inferior colliculus (CNIC), the basolateral amygdala (BLA) and the hippocampus. All three regions participate in sound processing differently. The CNIC, located at the posterior surface of the brainstem, is a channel for almost all auditory signals and the site of auditory signal integration, frequency recognition and pitch discrimination [12]. The BLA, as responsible for emotional processing, plays a valuable role in auditory fear conditioning, the regulation of acoustic startle response and the modulation of auditory cortex plasticity [13, 14]. The BLA also integrates acoustic inputs with the information from other senses and organisms′ internal state [15]. The “hearing hippocampus” [16] has wide auditory interactions, which is consistent with its extensive contributions to perception [16, 17]. Finally, a model has been proposed in which the limbic and auditory systems interact with one another during auditory disorder [18]. Within a few hours after exposure to loud noise there is elevated c‐fos expression in a various brain areas, including the limbic brain regions regulating emotion and attention (amygdala, nucleus accumbens, and lateral septum) [19–21]. The increase in transcription factor—fos should evoke changes in down‐stream gene expression.

In the present study in the hippocampus, BLA and CNIC regions of normal and noise‐exposed rats, we evaluated the levels of the following proteins: (i) involved in synaptic exocytosis—synaptophysin, syntaxin1, and SNAP‐25 [22, 23]; (ii) associated with oxidative stress and inflammatory processes—AIM2 and NLRP; (iii) implicated in oxidative phosphorylation; and (iv) malondialdehyde (MDA)‐protein adducts—markers of lipid peroxidation [24, 25]. In addition, using transmission electron microscopy (TEM), in abovementioned regions, we evaluated the consequences of noise on subcellular organization of different types of neurons, glial cells, and synapses. Based on our current information this is the first comparative study of ultrastructural and biochemical alterations induced by chronic loud noise in the classical auditory and limbic regions of the brain.

## 2. Materials and Methods

### 2.1. Animals and Experimental Design

Twenty‐four adult male Wistar rats (P137–142; weight ‐110–115) were used. The animals were provided by the Animal Center of Ivane Beritashvili Center of the Experimental Biomedicine. Before and during the experiments, the animals were maintained in wire‐meshed cages, three per cage, in a climate controlled space (20°C−22°C; 12 h light/dark cycle; humidity 55%–60%). Standard food pellets and tap water were available ad libitum. Just before the exposure to noise, the animals were randomly divided into experimental (*n* = 12) and control groups (*n* = 12). Experimental rats were repeatedly exposed to loud white noise. The control animals were untreated rats. For the biochemical studies, up to eight animals from each group (16 animals total) were used, and for the EM analysis—four animals from each group (eight animals total) were used. The research was carried out in accordance with the guidelines formulated by the European Council. The protocol received approval from the Ethics Committee of the Ilia State University as well as the Animal Care Committee of the Ivane Beritashvili Center of Experimental Biomedicine; Protocol Number: N01/10.02.2025.

### 2.2. Noise Exposure

In general, the process of noise exposure was the same as described in our earlier studies. The difference was only in the number of days of the noise exposure: experimental rats were subjected to 100 dB a high decibel level, for 30 consecutive days (1 h per day), instead of 10 days as in our previous studies [26–28]. According to the National Institute on Deafness and Other Communication Disorders noise at or below 70 dB, even after chronic exposure, is less likely to cause auditory pathology. However, long‐term exposure to sounds at or above 85 dB usually causes hearing damage. Chronic noise at 100–115 dB affects different elements of the cochlea. In particular, cochlear synaptopathy is one of the pathologies, that develops in the brain in response to chronic loud noise, characterized by the irreparable loss of synapses between inner hair cells and spiral ganglion neurons despite normal hearing thresholds [29–32]. In our study, the noise had a spectrum of 12–20 kHz, which falls within the range of human hearing and is prevalent in the production environments of manufacturing industries. Clinical and experimental studies suggested that this level of noise exposure provokes a number of detrimental effects, including cognitive disturbances [31–34].

For noise exposure, two Paradigm Signature S1 P‐Be loudspeakers (Paradigm Electronics Inc., Canada) were used. Four speakers were located 55 cm above the floor of the cages with experimental rats. Each speaker affected two cages. The level of noise was monitored continuously using two microphones, suspended in a line 45 cm above the cage.

The exposure to noise was performed in the daytime between 11.00 and 13.00. The biochemical and EM studies were conducted immediately after 30 days of noise exposure, in the daytime: 12.00–14.00.

### 2.3. Biochemical Analysis

The biochemical experiments were conducted separately on the CNIC, hippocampus, and BLA brain regions isolated from a group of eight male control and eight noise‐exposed rats (synaptophysin, SNAP25, and syntaxin studies), whereas for other biochemical studies each group consisted of six animals. The unanesthetized animals were killed by decapitation and the regions of interest were identified [35]. The dissected tissue samples were stored at −70°C until processing.

#### 2.3.1. Fraction Preparation

Frozen tissue samples were rapidly homogenized in a buffer of the following composition: 20 mM Tris‐HCl (pH 7.4), 0.32 M sucrose, 1 mM sodium orthovanadate, 1 mM ethylendiamintetraacetic acid, 0.5 mM ethylene glycol‐bis (2‐aminoethylether)‐N,N,N ^′^,N ^′^‐tetraacetic acid, 10 mM sodium pyrophosphate, and a cocktail of protease inhibitors (Sigma, P8340). A concentrated solution of sodium dodecyl sulfate (SDS) was added to the whole homogenate fraction to give a final concentration of 5%, which was heated at 95°C for 5 min.

#### 2.3.2. Protein Determination

Protein concentrations for each individual sample were measured in four technical replicates using a micro bicinchoninic acid (BCA) protein assay (Pierce/ThermoFisher Scientific, Cat.N. 23235).

#### 2.3.3. One‐Dimensional Electrophoresis and Western Blotting

**Figure 1:**
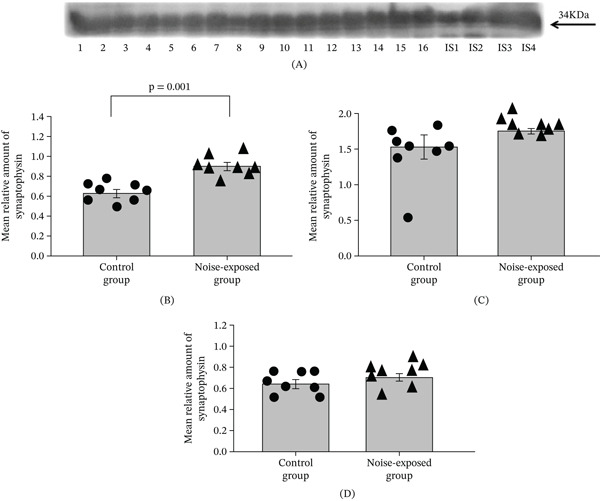
Western blot image (A, hippocampus) and (B) mean relative amounts ± sem of synaptophysin in the hippocampus, (C) BLA, and (D) CNIC of the control and noise‐exposed groups of male Wistar rats. Film contains 16 experimental samples; A: Lanes 1–8 control group; Lanes 9–16 white noise group. IS1–IS4 internal standards, containing 15, 30, 45, and 60 *μ*g protein corresponding to 0.5, 1.0, 1.5, and 2.0 relative amounts of protein. Individual values for each animal are shown on each bar. The difference for the hippocampus is significant (*p* = 0.001).

**Figure 2:**
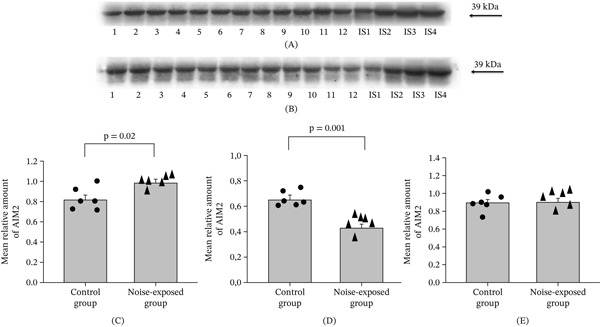
Western blot image (A‐CNIC and B‐BLA) and mean relative amounts ± sem of AIM2 in the (C) CNIC, (D) BLA, and (E) hippocampus of the control and noise‐exposed groups of male Wistar rats. The films contain 12 experimental samples: A: Lanes 1–6 control group; Lanes 7–12 white noise exposed group. IS1–IS4 internal standards, containing 15, 30, 45, and 60 *μ*g of protein corresponding to 0.5, 1.0, 1.5, and 2.0 relative amounts of protein. Individual values for each animal are shown on each bar. The differences are significant for CNIC (*p* = 0.02) and BLA (*p* = 0.0001).

**Figure 3:**
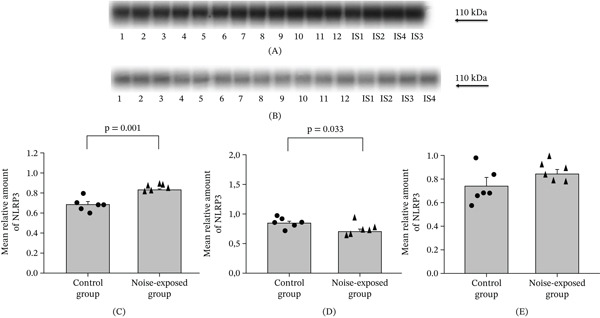
Western blot image (A‐CNIC and B‐BLA) and mean relative amounts ± sem of NLRP3 in the (C) CNIC, (D) BLA, and (E) hippocampus of the control and noise‐exposed groups of male Wistar rats (B). The film contains 12 experimental samples: A: Lanes 1–6 control group; Lanes 7–12 white noise exposed group. IS1–IS4 internal standards, containing 15, 30, 45, and 60 *μ*g protein corresponding to 0.5, 1.0, 1.5, and 2.0 relative amounts of protein. Individual values for each animal are shown on each bar. The differences are significant for CNIC (*p* = 0.001) and BLA (*p* = 0.033).

**Figure 4:**
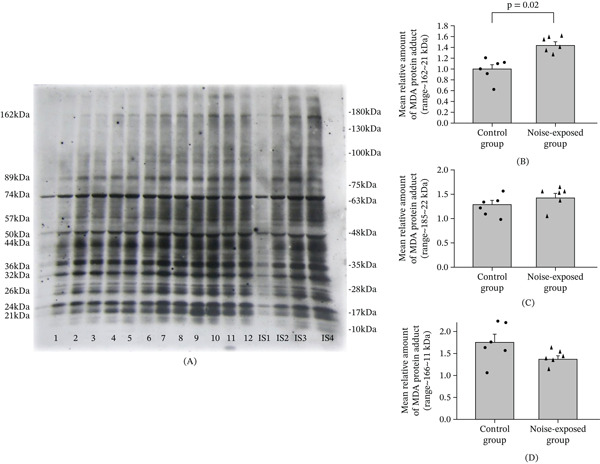
Western blot image (A‐CNIC) and mean relative amounts ± sem of MDA protein adducts in the (B) CNIC, (C) BLA, and (D) hippocampus in the noise‐exposed and control groups of male Wistar rats. The film contains 12 experimental samples: A: Lanes 1–6 control group; Lanes 7–12 white noise exposed group. IS1–IS4 internal standards, containing 15, 30, 45, and 60 *μ*g protein corresponding to 0.5, 1.0, 1.5, and 2.0 relative amounts of protein. On the left side of the film, the molecular weights of the major MDA protein adducts are indicated, whereas the right side shows the positions of the molecular weight standards. Individual values for each animal are shown on each bar. The difference for CNIC is significant (*p* = 0.002).

**Figure 5:**
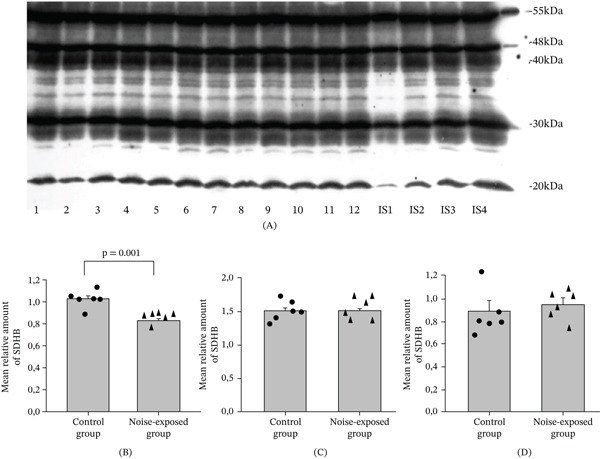
Expression of selected representative subunits of the mitochondrial OXPHOS system and ATP5A in the hippocampal region of the control and noise‐exposed rats. (A) Representative image of Western immunoblot. ATP5A‐55 kDa, UQCRC2‐48 kDa, MTCO1‐40 kDa, SDHB‐30 kDa, and NDUFB8‐20 kDa: Lanes 1–6 control group; Lanes 7–12 noise exposed group. IS1–IS4 internal standards, containing 15, 30, 45, and 60 *μ*g protein corresponding to 0.5, 1.0, 1.5, and 2.0 relative amounts of protein. (B) The mean amounts ± sem of SDHB in the hippocampus. The difference is significant (*p* = 0.001). The differences for SDHB in (C) BLA and in (D) CNIC are not significant. Individual values for each animal are shown on each bar.

Aliquots containing 30 *μ*g of total protein in 30 *μ*L of SDS sample buffer were used for SDS gel electrophoresis and Western blotting [36]. Proteins were transferred from polyacrylamide gels to nitrocellulose membranes, which were subsequently stained with the Ponceau S solution 0.1% (*w*/*v*). To verify efficient transfer and uniform gel loading the stained membranes were next digitized and analyzed using LabWorks 4.0 (UVP). The images of the Ponceau S stained membranes are provided in the Supporting Information (Figures S1–S18). The membranes were destained with PBS + 0.05*%* Tween 20. Where the difference between the molecular weights of the target proteins allowed simultaneous treatment of nitrocellulose membranes with different antibodies the membranes were cut according to the molecular weight standards in different parts and treated separately. Standard immunochemical staining was performed using peroxidase labeled secondary antibodies and the Super‐Signal West Pico Chemiluminescent substrate (Pierce, 34580). To ensure the linearity of signal, the nitrocellulose membranes were exposed to x‐ray films with intensifying screens. The films were preflashed with Sensitize (RPN2051, Amersham, Amersham, United Kingdom). The following antibodies were used as follows: (1) for the quantitation of components of the oxidative phosphorylation system (OXPHOS), total OXPHOS rodent WB antibody cocktail (ab110413, Abcam, United Kingdom, RRID: AB_2629281), (2) anti‐NLRP3 antibody [EPR23094‐1] (ab263899, Abcam, United Kingdom, RRID: AB_2629281), (3) anti‐AIM2 antibody (ab119791, Abcam, UK, RRID:AB_10899831), (4) anti‐MDA antibody [11E3] (ab243066, Abcam, United Kingdom, RRID:AB_3662056), (5) antisynaptophysin (ab32127, Abcam, United Kingdom, RRID:AB_228694), (6) anti‐SNAP25 (ab109105, Abcam, United Kingdom, RRID:AB_2286949), (7) antisyntaxin 1a (ab41453, Abcam, United Kingdom, RRID:AB_956343). The optical density of the respective protein bands was measured using LabWorks 4.0 (UVP). For the calibration of the autoradiographs, we included four internal protein standards in each of the gels. These standards comprise the homogenate fraction isolated from the brain of control rats. The same standards were used in all the experiments; they contained 15, 30, 45, and 60 *μ*g of total protein, respectively. For all the studied proteins and in all brain regions optical densities were directly proportional to the amount of loaded proteins (Figures S19–S33). The corresponding measures (Figures 1, 2, 3, 4, and 5) were obtained by dividing the individual optical band densities of a given sample (e.g., amygdala of noise‐treated animals) by the optical density, which, from the calibration of the same autoradiograph, corresponded to 30 *μ*g of total protein in the standard. The data obtained by this normalization are referred as the “relative amount” of protein. These densities of the respective protein bands were not normalized to any cellular housekeeping proteins, such as actin, since it cannot be assumed that such reference proteins remain unchanged under experimental conditions. Accordingly, to rule out any unreliability that might arise from normalization to housekeeping proteins [36, 37], we consistently validated the proper sample loading via Ponceau S staining (Figures S1–S18) as well as by the calibration with the mentioned protein standards.

### 2.4. TEM Study: Perfusion and Brain Processing

TEM studies were conducted immediately after 30 days of noise exposure. From each group, four animals were evaluated. For perfusion and brain processing, the routine protocol, applied in our laboratory was used [26]. Briefly, following intraperitoneal injection of pentobarbital (100 mg/kg, IP), the rats underwent transcardiac perfusion with 0.9% NaCl, followed by 500 mL of 4% paraphormaldehyde (0.1 M sodium cacodylate buffer, pH‐ 7.2–7.4) and 2.5% glutaraldehyde in 0.1 M PB, pH—7.4, at a perfusion pressure 120 mm Hg. The brains were removed from the skull and placed in the same fixative overnight. The left hemispheric tissue blocks containing areas of interest were cut into 400 micron‐thick coronal slices, washed in 0.1 M PB, and postfixed in 1% osmium tetroxide (0.1 M sodium cacodylate buffer, pH‐ 7.2–7.4) for 2 h. The CNIC, BLA, and CA1 area were identified using an optical microscope Leica MM AF, cut out from the slices, dehydrated in graded series of ethanol and acetone and embedded in araldite. From araldite blocks, 70–75 nm thick sections were prepared using an ultramicrotome Leica EM UC7. Ultrathin sections were mounted on 200‐mesh copper grids, double‐contrasted with uranyl‐acetate and lead citrate and observed using electron transmission microscope JEM 1400 (JEOL, Japan). From each rat, every fifth section, (10 sections total) was evaluated.

### 2.5. Statistical Analysis—Biochemical Study

The data for the CNIC, BLA, and the hippocampal area were analyzed separately. The details of the power calculation for the biochemical studies are provided in File S1 (Table S1). The unpaired Student′s *t*‐test was used for comparisons between two groups. All statistical tests conducted were two‐tailed, and the differences reaching a significance level of at least *p* < 0.05 were reported. Throughout the manuscript, data on the figures are reported as the mean ± standard error of the mean (sem) and individual values of each animal are also depicted.

## 3. Results

### 3.1. Immunoblotting

Detailed information about the antibodies′ reactivity, the internal standards, and regression plots are provided in File S2; “Immunoblotting” and Figures S19–S34.

#### 3.1.1. Proteins Involved in Synaptic Vesicle Exocytosis—Long‐Term Exposure to Loud Noise Induces Upregulation of Synaptophysin in the Hippocampal Region

##### 3.1.1.1. Synaptophysin.

In the hippocampus the mean amount of synaptophysin was significantly higher in the noise‐exposed group than in the control (*T* = 4.79, *p* = 0.001, DF = 14, Figure 1A,B). For CNIC and BLA no differences were significant (see Figure 1C,D and Figures S21 and S22, and File S3 [Table S2]).

##### 3.1.1.2. SNAP25 and Syntaxin.

No significant differences were observed for either protein in the brain regions studied (Figures S23–S28, Files S4 and S5 [Tables S3 and S4]).

### 3.2. Proteins Involved in Neuroinflammation; Long‐Term Exposure to Loud Noise Increases the Level of These Proteins in the CNIC and Decreases Them in the BLA

#### 3.2.1. AIM2

##### 3.2.1.1. CNIC.

The level of the AIM2 in the noise‐exposed group was significantly higher than in the control (*T* = 2.77, *p* = 0.020, DF = 10, Figure 2A,C).

##### 3.2.1.2. BLA.

The results, here, were opposite to those in the CNIC: The AIM2 protein level in the BLA was lower in the noise‐exposed group than the control (*T* = 86, *p* = 0.001, DF = 10, Figure 2B,D).

##### 3.2.1.3. The Hippocampal Area.

No differences were detected for AIM2 in the hippocampal area (Figure 2E, Figure S29, File S6 [Table S5]).

#### 3.2.2. NLRP3

##### 3.2.2.1. CNIC.

In noise‐exposed group, the level of NLRP3 was significantly higher than in the control (*T* = 4.63, *p* = 0.001, DF = 10, Figure 3A,C).

##### 3.2.2.2. BLA.

The level of NLRP in the BLA was higher in the control group than in the noise exposed group (*T* = 2.48, *p* = 0.033, DF = 10, Figure 3B,D).

##### 3.2.2.3. The Hippocampus.

No significant differences were revealed in the hippocampal region (Figure 3E, Figure S30, File S6 [Table S5]).

In conclusion, the proteins involved in neuroinflammation (components of inflammasome) are significantly changed after chronic noise exposure. These changes are opposite in the CNIC and BLA: The level of proteins was increased after noise exposure in the CNIC and decreased in the BLA.

### 3.3. Long‐Term Exposure to Loud Noise Increases the Level of MDA‐Protein Adducts in CNIC

In all three regions, several major and minor MDA‐protein adducts were identified (Figure 4, Figures S31 and S32). Some of the major MDA‐protein adducts, according to molecular range were similar between the brain regions (e.g., 42–44, 32, 26, 22–24 kDa protein bands).

#### 3.3.1. CNIC

The total amount of MDA‐protein adducts in the noise‐exposed group was significantly higher than the control (*T* = 4.03, *p* = 0.002, DF = 10, Figure 4). Separate analysis of major MDA‐protein adducts showed that the MDA‐protein adducts with the molecular weights 162, 89, 57, 50, 44, 26, 24, and 21 kDa were significantly increased in the CNIC of noise‐exposed animals as compared with the control (Figure 4A,B, File S7 [Table S6]). No significant differences were found for BLA (Figure 4C) and for hippocampus (Figure 4D).

### 3.4. Long‐Term Exposure to Loud Noise Affects the Level of Proteins Involved in Oxidative Phosphorylation and ATP Synthase FoF1 Complex ‐ Deficiency of Succinate Dehydrogenase Complex in the Hippocampal Area of Noise‐Exposed Rats

Using a targeted approach, we inquired whether there were any differences in the relative expression levels of respiratory chain Complexes I, II, III, and IV or the ATP synthase FoF1 complex in the abovementioned brain regions of the noise‐exposed and control groups of rats. Using a special antibody cocktail the following proteins were quantitatively assessed: (1) NADH: ubiquinone oxidoreductase subunit B8 (NDUFB8), Complex I; (2) succinate dehydrogenase complex iron sulfur subunit B (SDHB), Complex II; (3) ubiquinol‐cytochrome C reductase core protein 2 (UQCRC2, alternate name cytochrome B‐C1 complex Subunit 2), Complex III; (4) cytochrome c oxidase subunit 1 (MTCO1), Complex IV; (5) ATP synthase F1 subunit alpha (ATP5A). These proteins were selected because they are labile components of the corresponding complexes when they are assembled; thus, the measurement of these subunits allows for a detailed assessment of the relative levels of the individual complexes (https://www.abcam.com/total-oxphos-rodent-wb-antibody-cocktail-ab110413.html). The sample immunostaining pattern for the hippocampus is provided in Figure 5A. The sample immunostaining patterns for CNIC and BLA are presented on Figures S33 and S34.

#### 3.4.1. ATP5A, UQCRC2, MTCO1 and NDUFB8

No significant effects were detected in either brain region studied. No differences were observed between the groups.

#### 3.4.2. SDHB

There was a significant difference only in the hippocampus: The level of SDHB was significantly decreased in noise‐exposed rats (*T* = 6.28, *p* = 0.001, DF = 10, Figure 5B). In BLA and in CNIC significant differences by SDHB are not detected (Figure 5C,D).

### 3.5. EM Study; Long‐Term Exposure to Loud Noise Affects the Ultrastructure of CNIC, BLA and the Hippocampus

#### 3.5.1. CNIC

**Figure 6:**
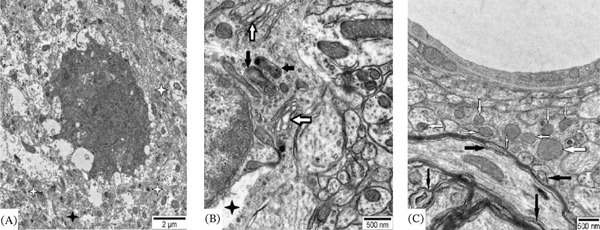
Representative electron micrographs of neuropil from the central nucleus of the inferior colliculus of adult male Wistar rat exposed to 30 days of chronic loud noise. (A) Apoptotic cell (white four‐point star); (B) fragment of neuron with electron lucent area (black four‐point star), mitochondria with condensed matrix (black arrows) and slightly moderately enlarged cisternae of Golgi apparatus (white arrows); (C) normal mitochondria (white arrows) and moderately demyelinated axon (black arrows).

In each group, 98 cells were analyzed. Compared with the control, in noise‐exposed animals, 16% of cells were damaged, namely medium‐ and large‐sized disk‐shaped neurons. In 9% of such cells, the modifications were moderate and included slightly diluted components of endoplasmic reticulum and Golgi complex, mitochondria with partially destructed cristae, mitochondria with condensed matrix or small myelin‐/membrane‐like cytoplasmic inclusions. In some neurons mild chromatolysis—reactive changes in the perikaryon of injured neurons, involving the rearrangement of rough endoplasmic reticulum and polyribosomes—were observed. In 7% of modified neurons substantial pathologies, such as apoptosis (identified by shrinkage of cells, nuclear fragmentation and phagocytosis by glial cells) (Figure 6A), central chromatolysis (injured cells, which may never again synthesize a normal level of proteins, or may atrophy and die) (Figure 6B), neurons with electron lucent cytoplasm and nucleus, significantly destroyed cytoplasmic organelles, large vacuoles, myelin‐like cytoplasmic inclusions, or even the signs of cell autophagy were identified. Presynaptic terminals of some axo‐dendritic synapses were swollen or contained slightly destructed mitochondria. Some astrocytes were somewhat swollen and contained small pathological inclusions, or slightly damaged cytoplasmic organelles. Rarely processes resembling dark microglia were observed. Compared with the control, demyelinated axons were relatively common (Figure 6C).

#### 3.5.2. BLA

**Figure 7:**
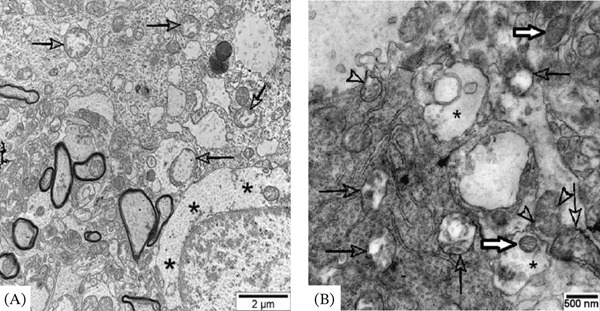
Representative electron micrographs of neuropil from the basolateral nucleus of the amygdala of an adult male Wistar rat exposed to 30 days of chronic loud noise. (A) Degenerate mitochondria (arrows) and neuronal chromatolysis (black asterisks); (B) altered mitochondria with disrupted cristae (black arrows), mitochondria with protrusions (black arrowheads), swollen presynaptic terminals (black asterisks), and normal mitochondria (white arrows).

In each group, 89 cells were analyzed. In noise‐exposed rats, approximately 11% of cells were modified, mostly pyramidal neurons. About 6% of these cells were significantly damaged. Specifically, cells with signs of apoptosis, neurons with destructed mitochondria, other damaged organelles and neurons with electron lucent cytoplasm and nucleus were found (Figure 7A). In others, moderate modifications (mild chromatolysis, moderate widening of components of endoplasmic apparatus and Golgi body, slightly damaged mitochondria, small vacuoles or small myelin/membrane‐like inclusions) were identified. Swollen presynaptic terminals and altered astrocytes were relatively rare (Figure 7B).

#### 3.5.3. Hippocampal CA1 region

Here, 97 cells in each group were analyzed. In noise‐exposed rats 8% cells were modified—mostly pyramidal neurons. However, substantial changes were detected only in 4% of these cells. The modifications were similar to those observed in CNIC and BLA but altered mitochondria were relatively numerous.

## 4. Discussion

Earlier, using the same experimental design and corresponding behavioral tests, namely, the basic open field, elevated plus maze, Morris water maze and the multibranch maze, we showed the alterations in anxiety activities and spatial memory in response to 10 days of high‐intensity chronic noise (100 dB) in male and female Wistar rats [26–28]. In addition, we reported that 10 days of intermittent exposures to chronic loud noise alters the ultrastructure of mitochondria in the medial geniculate body, considered as a key constituent of the classical auditory pathway, as well as in the hippocampus and amygdala, essential regions of nonclassical auditory pathway. Finally, the changes in the synaptic architecture (alterations in synaptic vesicle size and vesicle location) and porosome complex of classical auditory regions in response to 10 days′ exposure to high intensity white noise were described [27, 28, 38]. Our interest in the potential biochemical changes in these regions in response to noise was based on the abovementioned behavioral and ultrastructural data. In the present study, long‐lasting exposure to noise was used (30 days vs. 10 days). We suggest that chronic noise alters the level of proteins involved in neurotransmission, inflammation and oxidative stress. Due to the well‐known fact that continuous noise exposure has a more damaging effect than intermittent or relatively short‐term noise exposure and due to limited recovery time during continuous noise exposure, we did not perform additional behavioral studies.

Our biochemical and TEM data convincingly demonstrate region‐specific changes in proteins and morphology of the cells. In all brain areas, mostly principal/projection cells were damaged. Therefore, we propose that the main alterations develop in projective pathways, which bi‐directionally connect cortical and subcortical auditory and other structures. In the CNIC, BLA and the hippocampal CA1 area, nonspecific ultrastructural alterations that are usually observed in different types of neurological pathologies were detected. As expected, the most noticeable modifications were found in the classical component of auditory system, CNIC. The second region was BLA, limbic structure, which receives multiple sensory signals, including auditory stimuli.

Several data, including ours, point to morphological alterations in amygdala in response to loud noise [27, 39]. Moreover, it has been proposed that the noise exposure‐caused neurodegeneration of the amygdala in rats produces so‐called learned helplessness syndrome [39]. Finally, the most preserved was the ultrastructure of the hippocampus. Notably, according to recent data, Wistar rats exposed to 100 dBA for 12 h exhibit mainly molecular biological and biochemical alterations (immediate DNA fragmentation or long‐lasting neurotransmitter and immune‐histochemical modifications) [40].

Our TEM data indicate that 30 days of exposure to extensive noise produces more significant structural abnormalities than 10 days of noise [27]. This agrees with the hypothesis that the noise‐associated changes significantly depend on the duration of the noise exposure [41, 42].

Discussion of the data follows.

### 4.1. Gender Selection

A number of clinical and experimental studies, including ours, point to significant difference between males and females in noise‐exposed hearing loss. Males are at a higher risk of hearing loss than females, despite equivalent noise exposure and age [26, 28–30]. Earlier, we described gender differences in rats in the anxiety response and spatial memory to high intensity white noise [26, 28]; male were more vulnerable to noise than female. These differences must be taken into consideration when investigating the neurobiological components and/or treatment modalities. The clarification of the effects of high‐intensity chronic noise on specific protein levels in the brain of female rats is a subject of ongoing study.

### 4.2. Synaptic Vesicles

We studied quantitative changes in three proteins involved in different aspects of synaptic vesicle exocytosis and recycling: SNAP25, syntaxin 1, and synaptophysin. These proteins are known to be differently distributed between synaptic vesicles and synaptic plasma membranes: SNAP25 and syntaxin 1 are synaptic plasma membrane proteins, whereas synaptophysin is a major constituent of synaptic vesicular membranes, regardless of the identity of the neurotransmitter [43]. Significant alterations were revealed only for synaptophysin, which was increased in the hippocampal CA1 area of noise‐exposed animals. Selective change in only one studied protein among the three in the same biological pathways suggests for specific changes in the processes of neurotransmitter release. In general, biochemical findings suggest that chronic loud noise augments neurotransmission in the hippocampal CA1 area. Our early EM data also suggest the effect of noise on neurotransmission in this region. Specifically, using the same experimental paradigm, we noticed only a moderate effect of 10 days of intermittent loud noise on the ultrastructure of the hippocampus. However, measurable changes in different components of synapses, namely, a significant increase in the area of presynaptic mitochondria was noticed [38]. In the present study, relatively numerous swollen presynaptic terminals were detected in the hippocampus. These data may indicate altered neurotransmission. It is noteworthy that according to recent data, noise‐induced hearing loss is associated with changes in the gene expression in the cochlea. One of the candidate genes is SV2A—synaptic vesicle glycoprotein 2A, which is involved in the regulation of neurotransmitter release [44].

### 4.3. Neuroinflammation

According to our data, continuous loud noise significantly affects the expression of the inflammasome components. These effects vary in different brain regions. Namely, in the CNIC the expression of such components is increased, whereas in the BLA the expression is decreased. Generally, inflammasomes are considered as the central regulators of innate immunity, involved in the host defense against stress conditions [45]. Any significant deviation (upregulation or downregulation) from the normal levels of its components could be considered as pathological or compensatory conditions. Excessive or prolonged inflammasome activation can contribute to a damaging and destructive environment, resulting in the development of inflammatory disorders, including neurodegenerative diseases [45–48]. Based on the above‐mentioned mechanisms, we propose that prolonged exposure to loud noise results in long‐term inflammation and an increase in the components of inflammasome in the CNIC. Our studies of the increased level of MDA protein adduct in this zone support this view (see below).

In the BLA the opposite effect was observed; the components of the inflammasome were decreased. Different regulatory mechanisms and molecules may positively or negatively modulate the NLRP3 inflammasome. NEK7 (NIMA‐related kinase 7) is recruited for the formation of the NLRP3 inflammasome complex [46] and DDX3X (DEAD‐Box Helicase 3 X‐linked), GBP5 (Guanylate Binding protein 5), and cathepsin positively modulate the NLRP3 inflammasome [reviewed in 51]. At this step, we do not know which regulatory mechanism contributes to the decrease in inflammasome components.

In a recent study under an analogous experimental design, upregulation of the NLRP3 in the hippocampus was revealed [34]. Interestingly, the administration of the NLRP3 inhibitor MCC950 alleviated noise‐induced cognitive impairments in rats. Attenuation of noise‐induced hippocampal neuroinflammation, AD‐like pathologies, and neuronal damage was also observed [34].

The decrease in the components of inflammasome is known to have a beneficial effect on cell loss after status epilepticus (SE). For example, the knockdown of NLRP3 significantly decreases neuronal loss in the CA1 and CA3 areas of the hippocampus at 6 weeks after SE [49]. Therefore, the decrease in the AIM2 and NLRP3 in the BLA may be considered as a compensatory response, which could counteract the detrimental effects of noise in this area of the brain. Oppositely, the decrease in the activity could also be the result of regulatory failure. In conclusion, NLRP3 activity is a double‐edged sword; it is beneficial in some contexts but detrimental in others.

### 4.4. MDA‐Protein Adducts

Significant changes in MDA‐protein adducts were detected only in the CNIC. This parallels the upregulation of inflammasome components in the same region. Western blot analysis indicates that increased MDA‐protein adducts are detected for defined protein molecules but are not ubiquitous for all modified proteins. MDA may serve as a hapten to form neoantigens and evoke immunological response mechanisms. This process may represent a pathway by which lipid peroxidation could produce tissue damage via an immune mechanism [50]. Therefore, elevated MDA protein adducts in the CNIC after noise exposure may lead to increased neuroinflammation and inflammasome activity, which is documented by the observed expression of the inflammasome components—NLRP3 and AIM.

### 4.5. Mitochondria

From the brain regions studied, only in the hippocampal area were significant changes in OXPHOS levels detected via the panel of antibodies for the evaluation of OXPHOS levels. The expression of SDHB is decreased after exposure to noise. These changes indicate the decreased functioning of the succinate dehydrogenase complex. Recently it was shown that the inhibition of mitochondrial Complex II in neuronal cells leads to autophagy and neurodegeneration [51]. We cannot rule out the possibility of other changes in the mitochondrial proteome. In the hippocampus and neocortex of a mouse model of Rett syndrome, no changes were observed according to the panel of antibodies used in this study, though the levels of numerous mitochondrial proteins were altered [36]. The processes of synaptic plasticity including memory are associated with the increased expression of components of oxidative phosphorylation, other components of the mitochondrial proteome, and elevated mitochondrial dynamics [52–54]. EM analysis also showed altered mitochondria in all three regions, including the hippocampal CA1 area. Based on this finding, we propose that exposure to chronic loud noise could lead to the perturbation of synaptic plasticity processes via mitochondrial damage.

### 4.6. Possible Сrosstalk Between Neuroinflammation, Lipid Peroxidation and Mitochondrial

#### 4.6.1. Dysfunction After Chronic Exposure to Loud Noise Could Lead to Major Pathological Changes

Based on the biochemical results, we propose that, in addition to apoptosis [27, 55–59] one of the major consequences of prolonged loud noise in the defined brain regions may be ferroptosis. Ferroptosis, an iron‐dependent form of nonapoptotic cell death, is often associated with neurological disorders [60, 61]. Ferroptosis is closely links two pathological processes—oxidative stress and inflammatory responses, connected with iron and lipid metabolism [62, 63]. The activation of inflammation through the stimulation of multiple inflammation‐related signaling pathways, leads to ferroptosis [64]. The vulnerabilities of mitochondria might be particularly interesting as mitochondria associated ROS‐production and metabolic changes are mandatory for ferroptosis [65, 66]. Indeed, our biochemical results indicate a deficit in the SCDH complex after repeated loud noise exposure, which could lead to profound changes in mitochondrial metabolism. Our EM data might be another, indirect confirmation of the potential involvement of ferroptosis in noise‐induced pathologies. Namely, the most notable EM features of ferroptotic cells are the changes in mitochondrial morphology. For example, such cells typically often possess shrunken mitochondria with decreased membrane density [67], and different types of damaged mitochondria are often found in our material. Additional hallmarks of ferroptotic cells are electron lucent nucleus and cytoplasm, a possible defect of cytoplasm integrity [68, 69]. The same type of pathology is a common feature of necroptosis [68, 70]. However, another EM peculiarity of necroptosis, diluted perinuclear space, was not detected in our material. One more indirect indication of the involvement of ferroptosis could be the presence of the signs of autophagy [68, 69]. Recent studies indicate a link between autophagy and ferroptosis [65, 71, 72]. Moreover, the crosstalk between autophagy and ferroptosis is considered as a potential therapeutic target for auditory and some other pathologies. However, the mechanism of this interrelation remains to be determined [72–74]. The contribution of glia is crucial in mediating iron toxicity [53, 75]. However, glial reaction, observed in our material, could not be considered as a glial response to noise as it is common for different types of pathologies. Of special interest was the presence of dark processes, resembling “dark” microglia, in the classical auditory region, the CNIC. This type of microglia, a sign of cellular stress, is believed to be more active than “usual” microglia [75–79].

## 5. Conclusion

Most of the changes after chronic noise exposure suggest the pathological processes impairing the synaptic plasticity and normal functioning of the brain.

## 6. Significance

The combined biochemical and structural evaluation of alterations associated with chronic noise exposure provides important insights into the mechanisms by which this often underestimated environmental stressor contributes to physiological damage. These findings underscore the critical need for effective noise mitigation strategies to protect both mental and physical health. Detailed knowledge of the biological pathways and cellular changes implicated in the pathology of chronic loud noise action could pave the way to further translational research.

## 7. Limitations and Future Studies

Are the observed changes long‐lasting? Are these changes becoming stronger or disappearing over time? The absence of time‐dependent studies is the main limitation of our work and is the proposal for future studies.

## Author Contributions

Conceptualization: M.G.Z.; methodology: R.S. and M.G.Z; software: L.C. and L.T.; validation: N.J. and G.L.; formal analysis: N.P., L.C. and L.T.; investigation: F.R. and E.G.; resources: M.G.Z. and R.S.; data curation: N.P. and G.L.; writing—original draft preparation: M.G.Z. and R.S.; writing—review and editing: M.G.Z. and R.S.; visualization: F.R. and E.G.; supervision: M.G.Z. and R.S.; project administration: M.G.Z.; funding acquisition: M.G.Z.

## Funding

This study was supported by Shota Rustaveli National Science Foundation (10.13039/501100004801, FR‐21‐1501).

## Disclosure

Preprint of this paper was posted on https://www.preprints.org/manuscript/202505.1627. All authors have read and agreed to the published version of the manuscript.

## Ethics Statement

The protocol was approved by Committee of Ethics of Ilia State University and Animal Care Committee of Ivane Beritashvili Center of Experimental Biomedicine. Protocol Number: N01/10.02.2025.

## Consent

The authors have nothing to report.

## Conflicts of Interest

The authors declare no conflicts of interest.

## Supporting Information

Additional supporting information can be found online in the Supporting Information section.

## Supporting information


**Supporting Information 1** Figures S1–S18: Ponceau S stained image of all nitrocellulose membranes.

**Supporting Information 2** Figure S19: Calibration plots of optical density of internal standards with the amount of loaded protein for: (A) Synaptophysin, hippocampus; (B) AIM2 and CNIC; (C) AIM2 and BLA; and (D) NLRP3 and CNIC. Figure S20: Calibration plots of optical density of internal standards with the amount of loaded protein for: (A) NLRP3 and BLA; (B) MDA‐protein adducts and CNIC; and (C) SDHB and CA1. Figure S21: (A) Western blot image, (B) calibration plot, and (C) mean relative amounts ± sem of synaptophysin in BLA in control and noise‐exposed groups of male Wistar rats. Figure S22: (A) Western blot image, (B) calibration plot, and (C) mean relative amounts ± sem of Synaptophysin in CNIC in control and noise‐exposed groups of male Wistar rats. Figure S23: (A) Western blot image, (B) calibration plot, and (C) mean relative amounts ± sem of SNAP25 in CA1 in control and noise‐exposed groups of male Wistar rats. Figure S24: (A) Western blot image, (B) calibration plot, and (C) mean relative amounts ± sem of SNAP25 in BLA in control and noise‐exposed groups of male Wistar rats. Figure S25: (A) Western blot image, (B) calibration plot, and (C) mean relative amounts ± sem of SNAP25 in CNIC in control and noise‐exposed groups of male Wistar rats. Figure S26: (A) Western blot image, (B) calibration plot, and (C) mean relative amounts ± sem of syntaxin1a in CA1 in control and noise‐exposed groups of male Wistar rats. Figure S27: (A) Western blot image, (B) calibration plot, and (C) mean relative amounts ± sem of Syntaxin1a in BLA in control and noise‐exposed groups of male Wistar rats. Figure S28: (A) Western blot image, (B) calibration plot, and (C) mean relative amounts ± sem of syntaxin1a in CNIC in control and noise‐exposed groups of male Wistar rats. Figure S29: (A) Western blot image, (B) calibration plot, and (C) mean relative amounts ± sem of AIM2 in CA1 in control and noise‐exposed groups of male Wistar rats. Figure S30: (A) Western blot image, (B) calibration plot, and (C) mean relative amounts ± sem of NLRP3 in CA1 in control and noise‐exposed groups of male Wistar rats. Figure S31: (A) Western blot image, (B) calibration plot, and (C) mean relative amounts ± sem of MDA‐protein adducts in BLA in control and noise‐exposed groups of male Wistar rats. Figure S32: (A) Western blot image, (B) calibration plot, and (C) mean relative amounts ± sem of MDA‐protein adducts in CA1 in control and noise‐exposed groups of male Wistar rats. Figure S33: (A) Western blot image, (B) calibration plot, and (C) mean relative amounts ± sem of components of mitochondrial oxidative phosphorylation and ATP5A in CNIC in control and noise‐exposed groups of male Wistar rats. Figure S34: (A) Western blot image, (B) calibration plot, and (C) mean relative amounts ± sem of components of mitochondrial oxidative phosphorylation and ATP5A in BLA in control and noise‐exposed groups of male Wistar rats.

**Supporting Information 3** File S1 [Table S1]: Power calculation for sample size; File S2: Immunoblotting information; File S3 [TableS2]: Mean relative amounts of synaptophysin in CNIC, hippocampus, and BLA; File S4 [Table S3]: Mean relative amounts of SNAP25 in CNIC, hippocampus, and BLA; File S5 [Table S4]: Mean relative amounts of syntaxin in CNIC, hippocampus, and BLA; File S6 [Table S5]: Mean relative amounts of AIM2 and NLRP3 in CNIC, hippocampus, and BLA; File S7 [Table S6]: Mean relative amounts of different MDA‐protein adducts in IC.

## Data Availability

The original contributions presented in this study are included in the article/supporting information. Further inquiries can be directed to the corresponding author.

## References

[1] Krenz K. , Dhanani A. , McEachan R. R. C. , Sohal K. , Wright J. , and Vaughan L. , Linking the Urban Environment and Health: An Innovative Methodology for Measuring Individual-Level Environmental Exposures, International Journal of Environmental Research and Public Health. (2023) 20, no. 3, 10.3390/ijerph20031953. PMC9915172 36767317

[2] Le Prell C. G. , Clavier O. H. , and Bao J. , Noise-Induced Hearing Disorders: Clinical and Investigational Tools, Journal of the Acoustical Society of America. (2023) 153, no. 1, 711–722, 10.1121/10.0017002. 36732240 PMC9889121

[3] Kempen E. V. , Casas M. , Pershagen G. , and Foraster M. , WHO Environmental Noise Guidelines for the European Region: A Systematic Review on Environmental Noise and Cardiovascular and Metabolic Effects: A Summary, International Journal of Environmental Research and Public Health. (2018) 15, no. 2, 10.3390/ijerph15020379, 2-s2.0-85042465184. PMC5858448 29470452

[4] Münzel T. and Daiber A. , Vascular Redox Signaling, Endothelial Nitric Oxide Synthase Uncoupling, and Endothelial Dysfunction in the Setting of Transportation Noise Exposure or Chronic Treatment With Organic Nitrates, Antioxidants and Redox Signaling. (2013) 38, no. 13-15, 1001–1021, 10.1089/ars.2023.0006. PMC10171967 36719770

[5] Arjunan A. and Rajan R. , Noise and Brain, Physiology and Behavior. (2020) 227, 113136, 10.1016/j.physbeh.2020.113136. 32798569

[6] Cho I. , Kim J. , Jung S. , Kim S. Y. , Kim E. J. , Choo S. , Kam E. H. , and Koo B. N. , The Impact of Persistent Noise Exposure Under Inflammatory Conditions, Healthcare. (2023) 11, no. 14, 10.3390/healthcare11142067. PMC10379677 37510508

[7] Almasabi F. , van Zwieten G. , Alosaimi F. , Smit J. V. , Temel Y. , Janssen M. L. F. , and Jahanshahi A. , The Effect of Noise Trauma and Deep Brain Stimulation of the Medial Geniculate Body on Tissue Activity in the Auditory Pathway, Brain Sciences. (2022) 12, no. 8, 10.3390/brainsci12081099. PMC9405782 36009162

[8] Natarajan N. , Batts S. , and Stankovic K. M. , Noise-Induced Hearing Loss, Journal of Clinical Medicine. (2023) 12, no. 6, 10.3390/jcm12062347. PMC10059082 36983347

[9] Aime M. , Augusto E. , Kouskoff V. , Campelo T. , Martin C. , Humeau Y. , Chenouard N. , and Gambino F. , The Integration of Gaussian Noise by Long-Range Amygdala Ininputs in Frontal Circuit Promotes Fear Learning in Mice, Elife. (2020) 9, e62594, 10.7554/eLife.62594. 33252331 PMC7704104

[10] McLachlan N. M. and Wilson S. J. , The Contribution of Brainstem and Cerebellar Pathways to Auditory Recognition, Frontiers in Psychology. (2017) 20, no. 8, 10.3389/fpsyg.2017.00265, 2-s2.0-85016332434. PMC5357638 28373850

[11] Day H. E. , Masini C. V. , and Campeau S. , Reversible Inactivation of the Auditory Thalamus Disrupts HPA Axis Habituation to Repeated Loud Noise Stress Exposures, Brain Research. (2009) 1276, 123–130, 10.1016/j.brainres.2009.04.023, 2-s2.0-67349152391. 19379718 PMC2700553

[12] Oberle H. M. , Ford A. N. , Czarny J. E. , Rogalla M. M. , and Apostolidis P. F. , Recurrent Circuits Amplify Corticofugal Signals and Drive Feedforward Inhibition in the Inferior Colliculus, Journal of Neuroscience. (2023) 43, no. 31, 5642–5655, 10.1523/JNEUROSCI.0626-23.2023. 37308295 PMC10401644

[13] Gruene T. , Flick K. , Rendall S. , Cho J. H. , Gray J. , and Shansky R. , Activity-Dependent Structural Plasticity After Aversive Experiences in Amygdala and Auditory Cortex Pyramidal Neurons, Neuroscience. (2016) 328, 157–164, 10.1016/j.neuroscience.2016.04.045, 2-s2.0-84966709676. 27155146 PMC4888777

[14] Tempest G. D. and Parfitt G. , Prefrontal Oxygenation and the Acoustic Startle Eye Blink Response During Exercise: A test of the Dual-Mode Model, Psychophysiology. (2017) 54, no. 7, 1070–1080, 10.1111/psyp.12858, 2-s2.0-85016966393. 28370024

[15] Asokan M. M. , Watanabe Y. , Kimchi E. Y. , and Polley D. B. , Potentiation of Cholinergic and Corticofugal Inputs to the Lateral Amygdala in Threat Learning, Cell Reports. (2023) 42, no. 10, 10.1101/2023.01.31.526307. PMC10879743 37742187

[16] Billig A. J. , Lad M. , Sedley W. , and Griffiths T. D. , The Hearing Hippocampus, Progress in Neurobiology. (2022) 218, 102326, 10.1016/j.pneurobio.2022.102326. 35870677 PMC10510040

[17] Cusinato R. , Alnes S. L. , van Maren E. , Boccalaro I. , Ledergerber D. , Adamantidis A. , Imbach L. L. , Schindler K. , Baud M. O. , and Tzovara A. , Intrinsic Neural Timescales in the Temporal Lobe Support an Auditory Processing Hierarchy, Journal of Neuroscience. (2023) 43, no. 20, 3696–3707, 10.1523/JNEUROSCI.1941-22.2023. 37045604 PMC10198454

[18] Leaver A. M. , Seydell-Greenwald A. , and Rauschecker J. P. , Auditory-Limbic Interactions in Chronic Tinnitus: Challenges for Neuroimaging Research, Hearing Research. (2016) 334, 49–57, 10.1016/j.heares.2015.08.005, 2-s2.0-84940707421. 26299843 PMC7343340

[19] Wallhäusser-Franke E. , Mahlke C. , Oliva R. , Braun S. , Wenz G. , and Langner G. , Expression of c-fos in Auditory and Non-Auditory Brain Regions of the gerbil After Manipulations That Induce Tinnitus, Experimental Brain Research. (2003) 153, no. 4, 649–654, 10.1007/s00221-003-1614-2, 2-s2.0-0346788921. 14508632

[20] Palkovits M. , Helfferich F. , Dobolyi Á. , and Usdin T. B. , Acoustic Stress Activates Tuberoinfundibular Peptide of 39 Residues Neurons in the Rat Brain, Brain Structure and Function. (2009) 214, no. 1, 15–23, 10.1007/s00429-009-0233-5, 2-s2.0-72349086815. 19936783 PMC2819230

[21] Liu Y. , Alkharabsheh A. , and Sun W. , Hyperexcitability of the Nucleus Accumbens is Involved in Noise-Induced Hyperacusis, Neural Plasticity. (2020) 2020, no. 1, 8814858, 10.1155/2020/8814858. 33293947 PMC7714561

[22] Jena B. P. , Role of SNAREs in Membrane Fusion, Cell Fusion in Health and Disease. (2011) 713, 13–32, 10.1007/978-94-007-0763-4_3, 2-s2.0-79960962456. 21432012

[23] Rizo J. , Molecular Mechanisms Underlying Neurotransmitter Release, Annual Review of Biophysics. (2022) 51, no. 1, 377–408, 10.1146/annurev-biophys-111821-104732. PMC9490555 35167762

[24] Platnich J. M. and Muruve D. A. , NOD-Like Receptors and Inflammasomes: A Review of Their Canonical and Non-Canonical signaling Pathways, Archives of Biochemistry and Biophysics. (2019) 670, 4–14, 10.1016/j.abb.2019.02.008, 2-s2.0-85061808293. 30772258

[25] Wang W. , Zhang L. S. , Zinsmaier A. K. , Patterson G. , Leptich E. J. , Shoemaker S. L. , Yatskievych T. A. , Gibboni R. , Pace E. , Luo H. , Zhang J. , Yang S. , and Bao S. , Neuroinflammation Mediates Noise-Induced Synaptic Imbalance and Tinnitus in Rodent Models, Public Library of Science Biology. (2019) 17, no. 6, e3000307, 10.1371/journal.pbio.3000307, 2-s2.0-85068441705. PMC6581239 31211773

[26] Gogokhia N. , Japaridze N. , Tizabi Y. , Pataraya L. , and Zhvania M. G. , Gender Differences in Anxiety Response to High Intensity White Noise in Rats, Neuroscience Letters. (2021) 742, 135543, 10.1016/j.neulet.2020.135543. 33278506

[27] Zhvania M. , Gogokhia N. , Tizabi Y. , Japaridze N. , Pochkidze N. , Lomidze N. , Rzayev F. , and Gasimov E. , Behavioral and Neuroanatomical Effects on Exposure to White Noise in Rats, Neuroscience Letters. (2020) 728, 134898, 10.1016/j.neulet.2020.134898. 32224224

[28] Zhvania M. G. , Japaridze N. , Tizabi Y. , Pochkidze N. , and Lobzhanidze G. , Effects of High-Intensity Chronic Noise on Spatial Memory in Male Versus Female Rats, European Journal of Neuroscience. (2024) 60, no. 7, 5581–5590, 10.1111/ejn.16514. 39180282

[29] Xin J. , Shi Z. , Qian P. , Liu S. , Hao Y. , Gao X. , Zhou L. , Yang L. , and Zhang M. , Effectiveness of Kurtosis-Adjusted Cumulative Noise Exposure in Assessing Occupational Hearing Loss Associated With Complex Noise, Ear and Hearing. (2023) 44, no. 4, 865–876, 10.1097/AUD.0000000000001327. 36629325 PMC10262991

[30] Wang Q. , Wang X. , Yang L. , Han K. , Huang Z. , and Wu H. , Sex Differences in Noise-Induced Hearing Loss: A Cross-Sectional Study in China, Biology of Sex Differences. (2021) 12, no. 1, 10.1186/s13293-021-00369-0. PMC7937304 33676563

[31] Blum K. , Schepsky P. , Derleder P. , Schätzle P. , Nasri F. , Fischer P. , Engel J. , and Kurt S. , Noise-Induced Cochlear Synaptopathy in C57BL/6 N Mice as a Function of Trauma Strength: Ribbons Are More Vulnerable Than Postsynapses, Frontiers in Cellular Neuroscience. (2024) 18, 1465216, 10.3389/fncel.2024.1465216, 39411002. 39411002 PMC11473312

[32] Giraudet F. , Labanca L. , Souchal M. , and Avan P. , Decreased Reemerging Auditory Brainstem Responses Under Ipsilateral Broadband Masking as a Marker of Noise-Induced Cochlear Synaptopathy, Ear and Hearing. (2021) 42, no. 4, 1062–1071, 10.1097/AUD.0000000000001009, 33625059. 33625059

[33] Hu N. , Rutherford M. A. , and Green S. H. , Protection of Cochlear Synapses From Noise-Induced Excitotoxic Trauma by Blockade of Ca^2+^-Permeable AMPA Receptors, Proceedings of the National Academy of Sciences. (2020) 117, no. 7, 3828–3838, 10.1073/pnas.1914247117, 32015128. PMC7035499 32015128

[34] Ren Y. , Wu K. , He Y. , Zhang H. , Ma J. , Li C. , Ruan Y. , Zhang J. , Wen Y. , Wu X. , Chen S. , Qiu H. , Zhang Y. , Zhou L. , Ou Z. , Liang J. , and Wang Z. , The role of NLRP3 Inflammasome-Mediated Neuroinflammation in Chronic Noise-Induced Impairment of Learning and Memory Ability, Ecotoxicology and Environmental Safety. (2024) 286, 117183, 10.1016/j.ecoenv.2024.117183, 39437517. 39437517

[35] Paxinos G. and Watson C. , The Rat Brain in Stereotaxic Coordinates, 2006, Sixth edition, Elsevier. 10.1016/0165-0270(80)90021-7 6110810

[36] Golubiani G. , van Agen L. , Tsverava L. , Solomonia R. , and Müller M. , Mitochondrial Proteome Changes in Rett Syndrome, Biology. (2023) 12, no. 7, 10.3390/biology12070956. PMC10376342 37508386

[37] Dittmer A. and Dittmer J. , Beta-Actin Is Not a Reliable Loading Control in Western Blot Analysis, Electrophoresis. (2006) 27, no. 14, 2844–2845, 10.1002/elps.200500785, 2-s2.0-33746738785. 16688701

[38] Pochkhidze N. , Gogokhia N. , Japaridze N. , Lazrishvili I. , Bikashvili T. , and Zhvania M. G. , Electron Microscopy Demonstrating Noise Exposure Alters Synaptic Vesicle Size in the Inferiorcolliculus of Cat, Noise and Health. (2021) 23, no. 109, 51–56, 10.4103/nah.NAH_26_20. 34213447 PMC8411948

[39] Dumlu A. M. , Kanat A. , Aydin N. , Ozcan Tozoglu E. , Aydin Okuyan A. , Demirtas R. , and Kadioglu H. H. , New Finding for Neuronal Basis of the Learned Helplessness Syndrome Secondary to Loudness: Nonresistant Rats Have More Damaged Neurons in the Amygdala Than Resistant Rats, Neurological Research. (2025) 47, no. 11, 1034–1044, 10.1080/01616412.2025.2512132. 40491078

[40] Frenzilli G. , Ryskalin L. , Ferrucci M. , Cantafora E. , Chelazzi S. , Giorgi F. S. , Lenzi P. , Scarcelli V. , Frati A. , Biagioni F. , Gambardella S. , Falleni A. , and Fornai F. , Loud Noise Exposure Produces DNA, Neurotransmitter and Morphological Damage within Specific Brain Areas, Frontiers in Neuroanatomy. (2017) 11, 10.3389/fnana.2017.00049, 2-s2.0-85021412479. PMC5483448 28694773

[41] Li W. , Zhang H. , Xiao Y. , Tao Y. , Chen W. , and Wang D. , Association Between Occupational Noise Exposure Duration and Heart Rate Variability Among Chinese Adults: The Role of Plasma miRNAs, Environmental Pollution. (2023) 323, 121324, 10.1016/j.envpol.2023.121324. 36813098

[42] Rogers C. M. , Palmerton H. , Saway B. , Tomlinson D. , and Simonds G. , Effect of Various OR Noise on Fine Motor Skills, Cognition, and Mood, Surgery Research and Practice. (2019) 2019, no. 1, 5372174, 10.1155/2019/5372174. 31355326 PMC6637681

[43] Bera M. , Radhakrishnan A. , Coleman J. , Sundaram R. V. K. , Ramakrishnan S. , Pincet F. , and Rothman J. E. , Synaptophysin Chaperones the Assembly of 12 SNAREpins Under Each Ready-Release Vesicle, Proceedings of the National Academy of Sciences. (2023) 120, no. 45, e2311484120, 10.1073/pnas.2311484120. PMC10636311 37903271

[44] Lavinsky J. , Kasperbauer G. , Bento R. F. , Mendonca A. , Wang J. , Crow A. L. , Allayee H. , and Friedman R. A. , Noise Exposure and Distortion Product Otoacoustic Emission Suprathreshold Amplitudes: A Genome-Wide Association Study, Audiology and Neurotology. (2021) 26, no. 6, 445–453, 10.1159/000514143. 34280920 PMC8612944

[45] Voet S. , Srinivasan S. , Lamkanfi M. , and van Loo G. , Inflammasomes in Neuroinflammatory and Neurodegenerative Diseases, European Molecular Biology Organization Molecular Medicine. (2019) 11, no. 6, EMMM201810248, 10.15252/emmm.201810248, 2-s2.0-85064631879. PMC6554670 31015277

[46] Lamkanfi M. and Dixit V. M. , Inflammasomes and Their Roles in Health and Disease, Annual Review of Cell and Developmental Biology. (2012) 28, 137–161, 10.1146/annurev-cellbio-101011-155745, 2-s2.0-84869504451. 22974247

[47] McManus R. M. and Latz E. , NLRP3 Inflammasome Signalling in Alzheimer′s Disease, Neuropharmacology. (2024) 252, 109941, 10.1016/j.neuropharm.2024.109941. 38565393

[48] Singh J. , Habean M. L. , and Panicker N. , Inflammasome Assembly in Neurodegenerative Diseases, Trends in Neurosciences. (2023) 46, no. 10, 814–831, 10.1016/j.tins.2023.07.009. 37633753 PMC10530301

[49] Meng X. F. , Tan L. , Tan M. S. , Jiang T. , Tan C. C. , Li M. M. , Wang H. F. , and Yu J. T. , Inhibition of the NLRP3 Inflammasome Provides Neuroprotection in Rats Following Amygdala Kindling-Induced Status Epilepticus, Journal of Neuroinflammation. (2014) 11, no. 1, 10.1186/s12974-014-0212-5, 2-s2.0-84924911891. PMC4275944 25516224

[50] Liang P. , Zhang X. , Zhang Y. , Wu Y. , Song Y. , Wang X. , Chen T. , Liu W. , Peng B. , Yin J. , He F. , Fan Y. , Han S. , and He X. , Neurotoxic A1 Astrocytes Promote Neuronal Ferroptosis via CXCL10/CXCR3 Axis in Epilepsy, Free Radical Biology and Medicine. (2023) 195, 329–342, 10.1016/j.freeradbiomed.2023.01.002. 36610561

[51] Ranganayaki S. , Jamshidi N. , Aiyaz M. , Rashmi S. K. , Gayathri N. , Harsha P. K. , Padmanabhan B. , and Srinivas Bharath M. M. , Inhibition of Mitochondrial Complex II in Neuronal Cells Triggers Unique Pathways Culminating in Autophagy With Implications for Neurodegeneration, Scientific Reports. (2021) 11, no. 1, 10.1038/s41598-020-79339-2. PMC7810707 33452321

[52] Meparishvili M. , Nozadze M. , Margvelani G. , McCabe B. J. , and Solomonia R. O. , A Proteomic Study of Memory After Imprinting in the Domestic Chick, Frontiers in Behavioral Neuroscience. (2015) 9, 10.3389/fnbeh.2015.00319. PMC4660867 26635566

[53] Solomonia R. O. , Kunelauri N. , Mikautadze E. , Apkhazava D. , McCabe B. J. , and Horn G. , Mitochondrial Proteins, Learning and Memory: Biochemical Specialization of a Memory System, Neuroscience. (2011) 194, 112–123, 10.1016/j.neuroscience.2011.07.053, 2-s2.0-80053109035. 21839805

[54] Margvelani G. , Meparishvili M. , Tevdoradze E. , McCabe B. J. , and Solomonia R. , Mitochondrial Fusion and Fission Proteins and the Recognition Memory of Imprinting in Domestic Chicks, Neuroreport. (2018) 29, no. 2, 128–133, 10.1097/WNR.0000000000000936, 2-s2.0-85042378710. 29189636

[55] Chen D. , Jia G. , Zhang Y. , Mao H. , Zhao L. , Li W. , Chen Y. , and Ni Y. , Sox2 Overexpression Alleviates Noise-Induced Hearing Loss by Inhibiting Inflammation-Related Hair Cell Apoptosis, Journal of Neuroinflammation. (2022) 19, no. 1, 10.1186/s12974-022-02414-0. PMC8883703 35227273

[56] Op de Beeck K. , Schacht J. , and Van Camp G. , Apoptosis in Acquired and Genetic Hearing Impairment: the Programmed Death of the Hair Cell, Hearing Research. (2011) 281, no. 1-2, 18–27, 10.1016/j.heares.2011.07.002, 2-s2.0-80055114418. 21782914 PMC3341727

[57] Wu J. , Ye J. , Kong W. , Zhang S. , and Zheng Y. , Programmed Cell Death Pathways in Hearing Loss: A Review of Apoptosis, Autophagy and Programmed Necrosis, Cell Proliferation. (2020) 53, no. 11, e12915, 10.1111/cpr.12915. 33047870 PMC7653260

[58] Oshitari T. , Neurovascular Cell Death and Therapeutic Strategies for Diabetic Retinopathy, International Journal of Molecular Sciences. (2023) 24, no. 16, 12919, 10.3390/ijms241612919. 37629100 PMC10454228

[59] Tang D. , Ch X. , Kang R. , and Kroemer G. , Ferroptosis: Molecular Mechanisms and Health Implications, Cell Research. (2020) 31, no. 2, 107–125, 10.1038/s41422-020-00441-1. 33268902 PMC8026611

[60] Chen Y. , Fang Z. M. , Yi X. , Wei X. , and Jiang D. S. , The Interaction Between Ferroptosis and Inflammatory Signaling Pathways, Cell Death & Disease. (2023) 14, no. 3, 10.1038/s41419-023-05716-0. PMC10030804 36944609

[61] Liu S. , Gao X. , and Zhou S. , New Target for Prevention and Treatment of Neuroinflammation: Microglia Iron Accumulation and Ferroptosis, American Society for Neurochemistry Neuro. (2020) 14, no. 1, 17590914221133236, 10.1177/17590914221133236. PMC9607999 36285433

[62] Wang Z. L. , Yuan L. , Li W. , and Li J. Y. , Ferroptosis in Parkinson′s Disease: Glia-Neuron Crosstalk, Trends in Molecular Medicine. (2022) 28, no. 4, 258–269, 10.1016/j.molmed.2022.02.003. 35260343

[63] Dixon S. J. , Lemberg K. M. , Lamprecht M. R. , Skouta R. , Zaitsev E. M. , Gleason C. E. , Patel D. N. , Bauer A. J. , Cantley A. M. , Yang W. S. , Morrison B. , and Stockwell B. R. , Ferroptosis: An Iron-Dependent Form of Nonapoptotic Cell Death, Cell. (2012) 149, no. 5, 1060–1072, 10.1016/j.cell.2012.03.042, 2-s2.0-84861541814. 22632970 PMC3367386

[64] Costigan A. , Hollville E. , and Martin S. J. , Discriminating Between Apoptosis, Necrosis, Necroptosis, and Ferroptosis by Microscopy and Flow Cytometry, Current Protocols. (2023) 3, no. 12, e951, 10.1002/cpz1.951. 38112058

[65] Tan X. , He Y. , Yu P. , Deng Y. , Xie Z. , Guo J. , Hou Q. , Li P. , Lin X. , Ouyang S. , Ma W. , Xie Y. , Guo Z. , Chen D. , Zhang Z. , Zhu Y. , Huang F. , Zhao Z. , Zhang C. , Guo Z. , Chen X. , Peng T. , Li L. , and Xie W. , The dual role of FSP1 in Programmed Cell Death: Resisting Ferroptosis in the Cell Membrane and Promoting Necroptosis in the Nucleus of THP-1 cells, Molecular Medicine. (2024) 30, no. 1, 10.1186/s10020-024-00861-4. PMC11247902 39009982

[66] Miyake S. , Murai S. , Kakuta S. , Uchiyama Y. , and Nakano H. , Identification of the Hallmarks of Necroptosis and Ferroptosis by Transmission Electron Microscopy, Biochemical and Biophysical Research Communications. (2020) 527, no. 3, 839–844, 10.1016/j.bbrc.2020.04.127. 32430176

[67] Gao W. , Wang X. , Zhou Y. , Wang X. , and Yu Y. , Autophagy, Ferroptosis, Pyroptosis, and Necroptosis in Tumor Immunotherapy, Signal Transduction and Targeted Therapy. (2022) 7, no. 1, 10.1038/s41392-022-01046-3. PMC9208265 35725836

[68] Liu C. , Li Z. , Li B. , Liu W. , Zhang S. , Qiu K. , and Zhu W. , Relationship Between Ferroptosis and Mitophagy in Cardiac Ischemia Reperfusion Injury: A Mini-Review, PeerJ. (2023) 11, e14952, 10.7717/peerj.14952. 36935924 PMC10019339

[69] Jian B. , Pang J. , Xiong H. , Zhang W. , Zhan T. , Su Z. , Lin H. , Zhang H. , He W. , and Zheng Y. , Autophagy-Dependent Ferroptosis Contributes to Cisplatin-Induced Hearing Loss, Toxicology Letters. (2021) 350, 249–260, 10.1016/j.toxlet.2021.07.010. 34302894

[70] Sun Y. , Zou S. , He Z. , and Chen X. , The Role of Autophagy and Ferroptosis in Sensorineural Hearing Loss, Frontiers in Neuroscience. (2022) 16, 1068611, 10.3389/fnins.2022.1068611. 36578828 PMC9791179

[71] Flury A. , Aljayousi L. , Park H. J. , Khakpour M. , Mechler J. , Aziz S. , McGrath J. D. , Deme P. , Sandberg C. , González Ibáñez F. , Braniff O. , Ngo T. , Smith S. , Velez M. , Ramirez D. M. , Avnon-Klein D. , Murray J. W. , Liu J. , Parent M. , Mingote S. , Haughey N. J. , Werneburg S. , Tremblay M. È. , and Ayata P. , A Neurodegenerative Cellular Stress Response Linked to Dark Microglia and Toxic Lipid Secretion, Neuron. (2025) 113, no. 4, 554–571, 10.1016/j.neuron.2024.11.018. 39719704 PMC12481204

[72] St-Pierre M. K. , Bordeleau M. , and Tremblay M. È. , Visualizing Dark Microglia, Microglia: Methods and Protocols, 2019, Springer, 97–110, 10.1007/978-1-4939-9658-2_8, 2-s2.0-85071471255. 31392680

[73] Wang J. , He W. , and Zhang J. , A richer and More Diverse Future for Microglia Phenotypes, Heliyon. (2023) 9, no. 4, e14713, 10.1016/j.heliyon.2023.e14713. 37025898 PMC10070543

[74] Thompson R. , Smith R. B. , Bou Karim Y. , Shen C. , Drummond K. , Teng C. , and Toledano M. B. , Noise Pollution and Human Cognition: An Updated Systematic Review and Meta-Analysis of Recent Evidence, Environment International. (2022) 158, 106905, 10.1016/j.envint.2021.106905. 34649047

[75] Irrera N. , Russo M. , Pallio G. , Bitto A. , Mannino F. , Minutoli L. , Altavilla D. , and Squadrito F. , The role of NLRP3 Inflammasome in the Pathogenesis of Traumatic Brain Injury, International Journal of Molecular Sciences. (2020) 21, no. 17, 10.3390/ijms21176204. PMC7503761 32867310

[76] Guha L. , Singh N. , and Kumar H. , Different Ways to Die: Cell Death Pathways and Their Association With Spinal Cord Injury, Neurospine. (2023) 20, no. 2, 430–448, 10.14245/ns.2244976.488. 37401061 PMC10323345

[77] Hu X. , Xu Y. , Xu H. , Jin C. , Zhang H. , Su H. , Li Y. , Zhou K. , and Ni W. , Progress in Understanding Ferroptosis and Its Targeting for Therapeutic Benefits in Traumatic Brain and Spinal Cord Injuries, Frontiers in Cell and Developmental Biology. (2021) 9, 705786, 10.3389/fcell.2021.705786. 34422826 PMC8371332

[78] Tisato V. , Castiglione A. , Ciorba A. , Aimoni C. , Silva J. A. , Gallo I. , D′Aversa E. , Salvatori F. , Bianchini C. , Pelucchi S. , Secchiero P. , Zauli G. , Singh A. V. , and Gemmati D. , LINE-1 Global DNA Methylation, Iron Homeostasis Genes, Sex and Age in Sudden Sensorineural Hearing Loss (SSNHL), Human Genomics. (2023) 17, no. 1, 10.1186/s40246-023-00562-9. PMC10722762 38098073

[79] Blackwell J. M. , Lesicko A. M. , Rao W. , De Biasi M. , and Geffen M. N. , Auditory Cortex Shapes Sound Responses in the Inferior Colliculus, Elife. (2020) 9, e51890, 10.7554/eLife.51890. 32003747 PMC7062464

