# Supplementary material for: Chronic Loud Noise—Biochemical and Ultrastructural Alterations in Auditory and Limbic Regions of the Rat Brain
Source: Biomed Res Int. 2026 Apr 24;2026:2680036. doi: 10.1155/bmri/2680036 (PMC13108246; doi:10.1155/bmri/2680036)

Ponceau S Stained Images

Supplementary Figure S1. Ponceau S stained image of nitrocellulose membrane (Hippocampus) further used for Synaptophysin immunoblotting. Last lane MW standards. Four lanes before MW standards internals standards (15, 30,45 and 60 mkg of total protein)

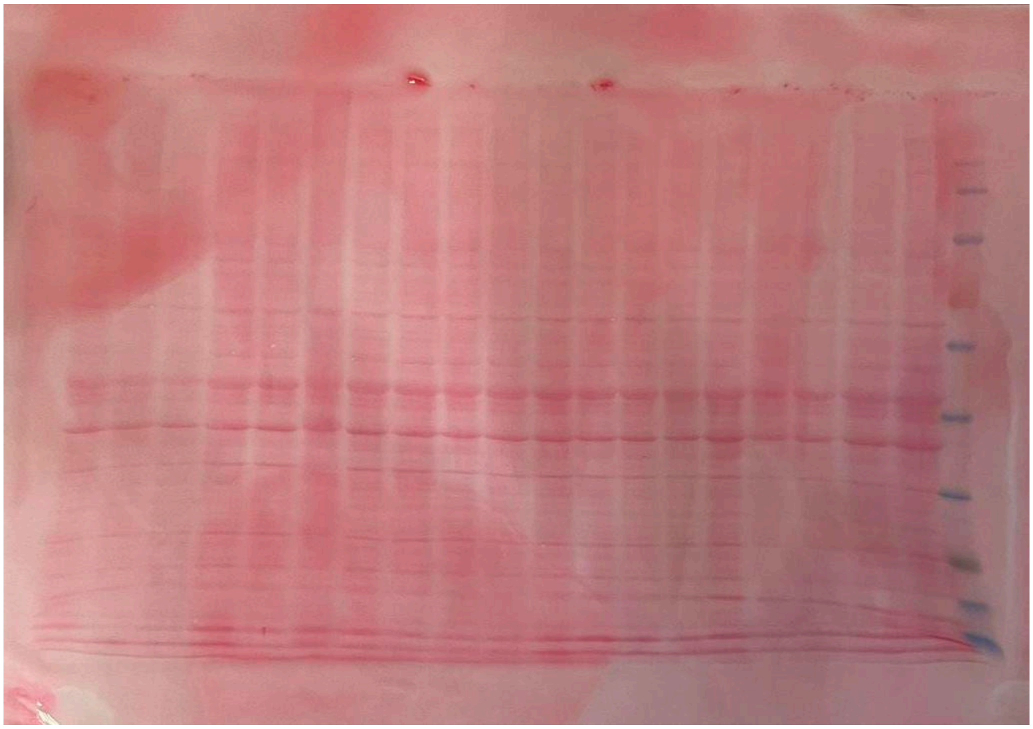

Supplementary Figure S2. Ponceau S stained image of nitrocellulose membrane (CNIC) further used for Synaptophysin immunoblotting. First lane MW standards. Four last lanes internals standards (15, 30,45 and 60 mkg of total protein)

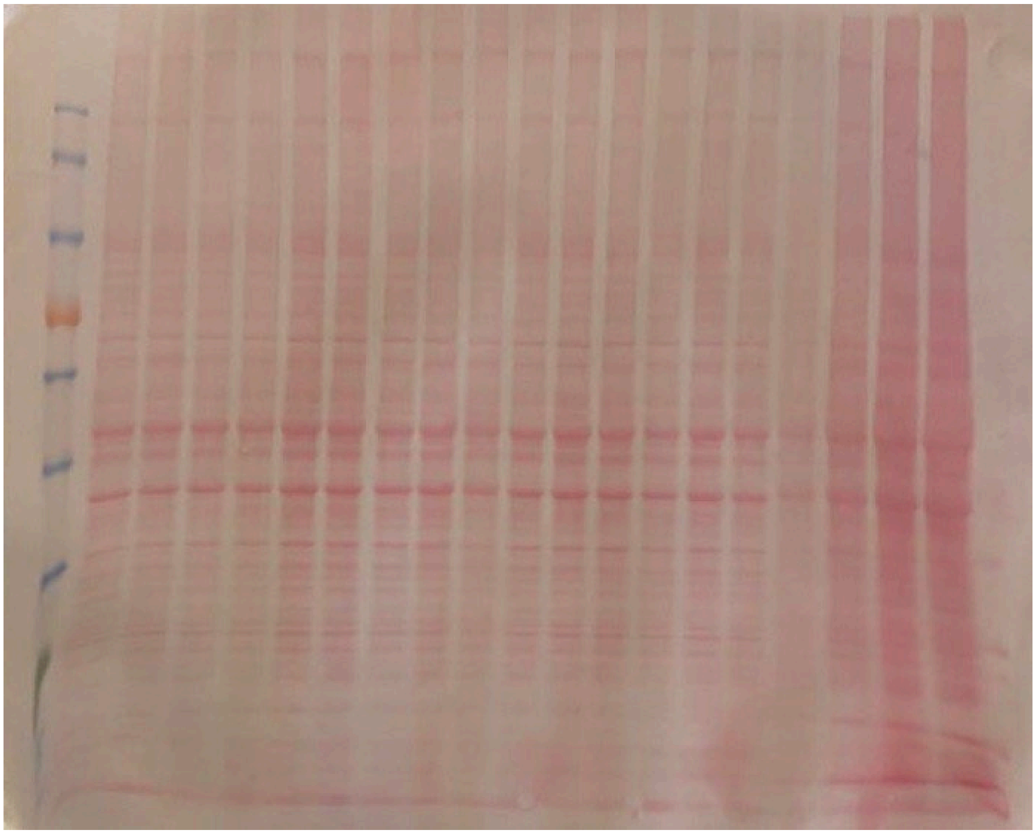

Supplementary Figure S3. Ponceau S stained image of nitrocellulose membrane (BLA) further used for Synaptophysin immunoblotting. Last lane MW standards. Four lanes before MW standards - internals standards (15, 30,45 and 60 mkg of total protein

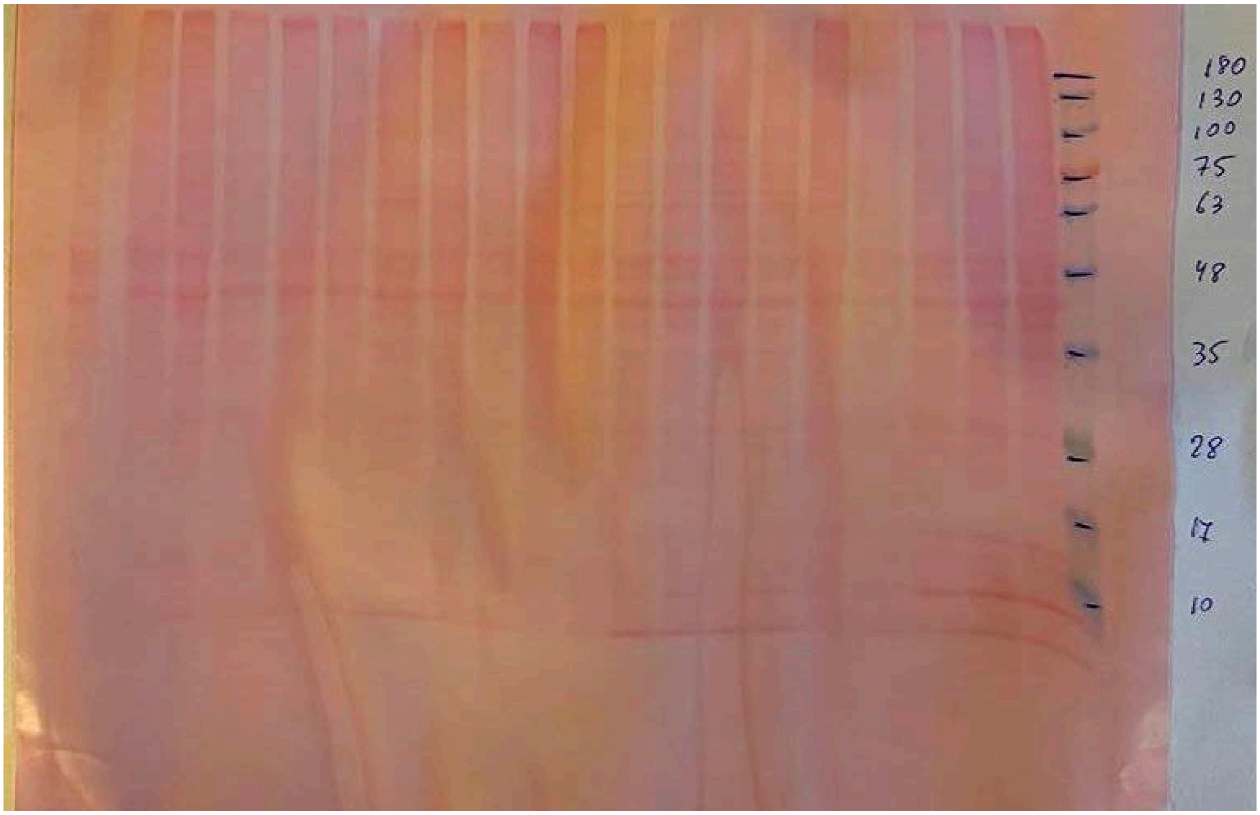

Supplementary Figure S4. Ponceau S stained image of nitrocellulose membrane (Hippocampus) further used for Syntaxin 1a immunoblotting. Last lane MW standards. Four lanes before MW standards - internals standards (15, 30,45 and 60 mkg of total protein.

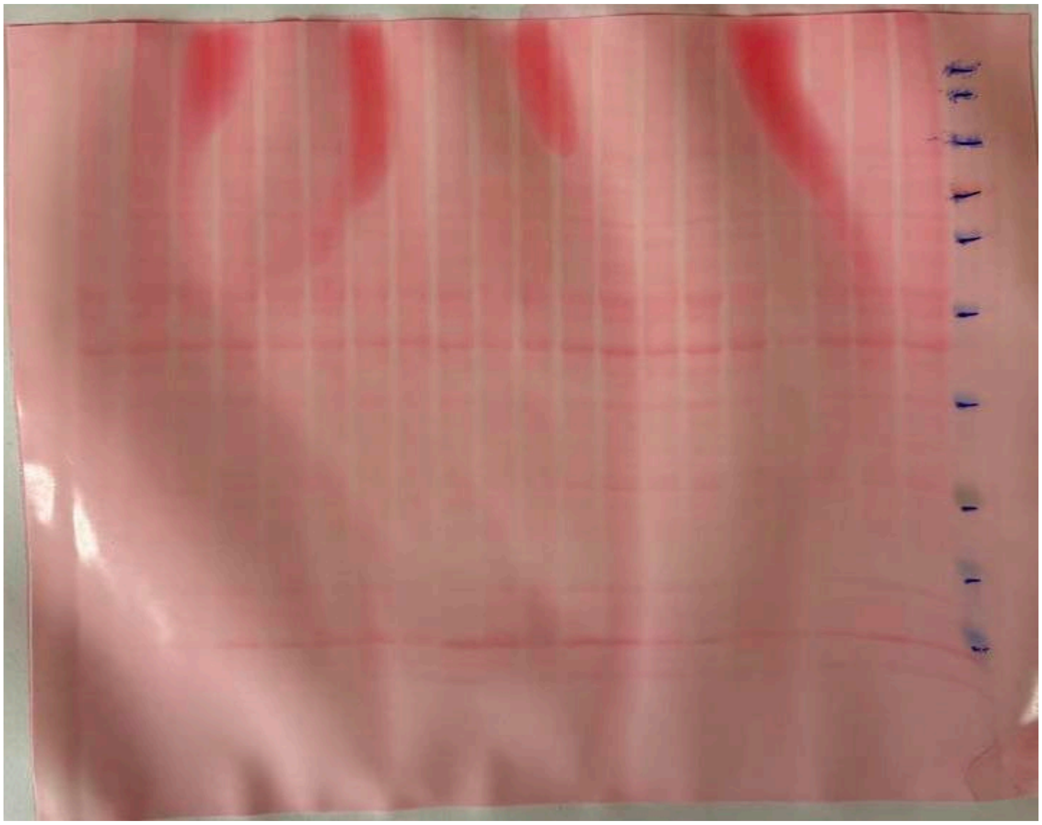

Supplementary Figure S5. Ponceau S stained image of nitrocellulose membrane (CNIC) further used for Syntaxin 1a immunoblotting. Four last lanes - internals standards (15, 30,45 and 60 mkg of total protein).

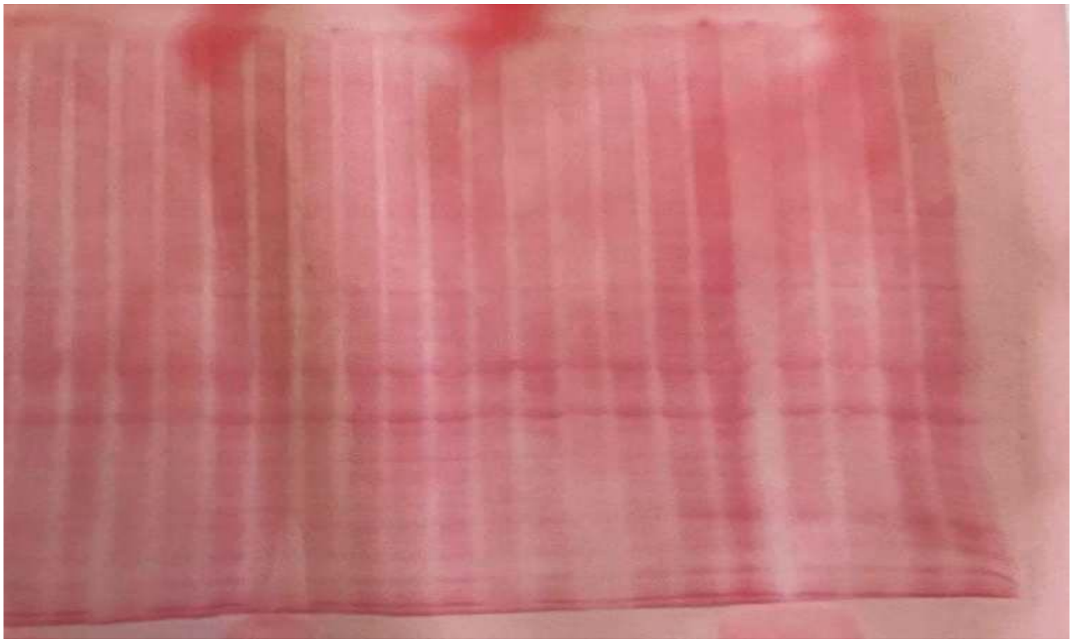

Supplementary Figure S6. Ponceau S stained image of nitrocellulose membrane (BLA) further used for Syntaxin 1a immunoblotting. Four last lanes - internals standards (15, 30,45 and 60 mkg of total protein. First lane MW standards.

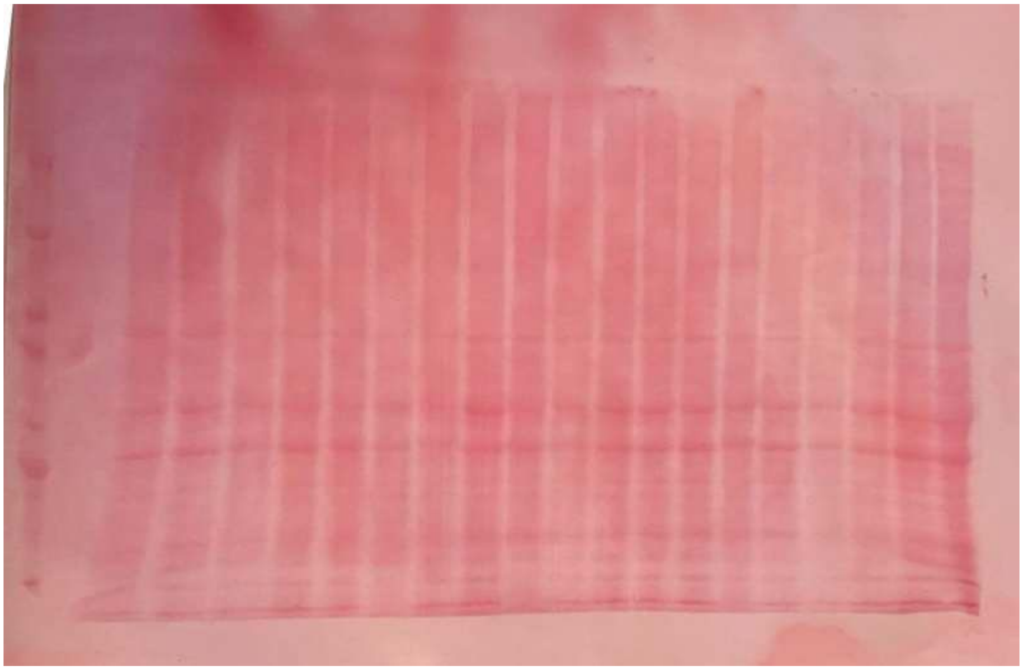

Supplementary Figure S7. Ponceau S stained image of nitrocellulose membrane (Hippocampus) further used for SNAP 25 immunoblotting. Last lane MW standards. Four lanes before last - internals standards (15, 30,45 and 60 mkg of total protein.

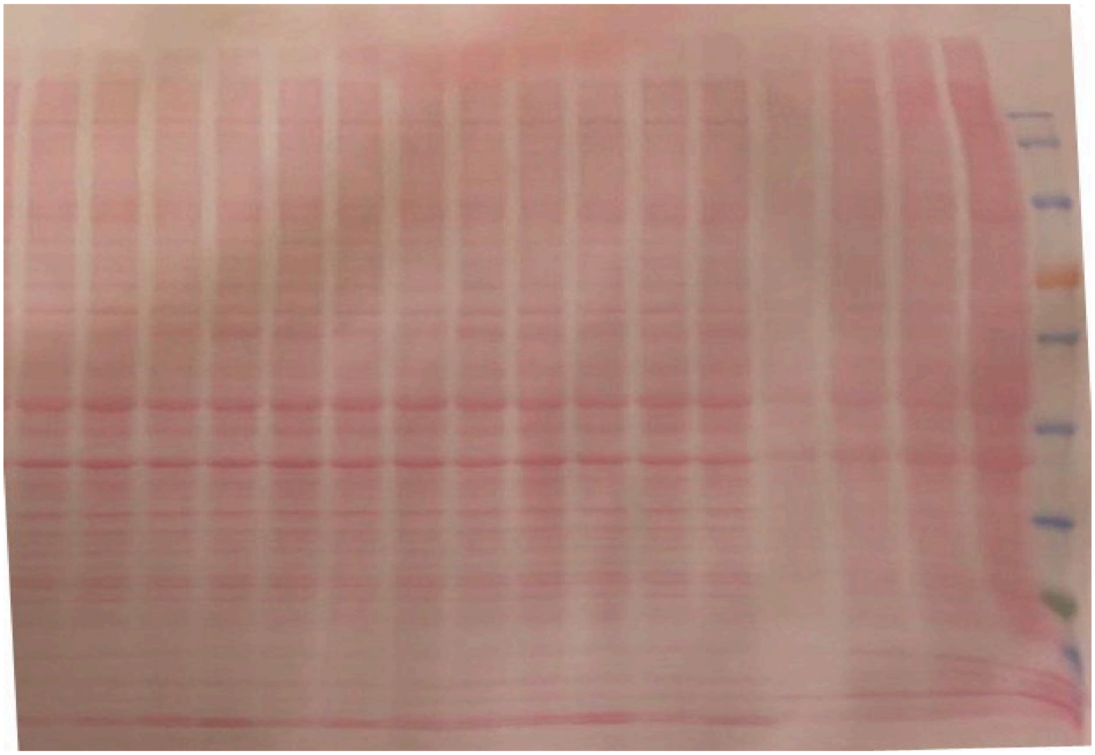

Supplementary Figure S8. Ponceau S stained image of nitrocellulose membrane (CNIC) further used for SNAP 25 immunoblotting. Last lane MW standards. Four lanes before last - internals standards (15, 30,45 and 60 mkg of total protein.

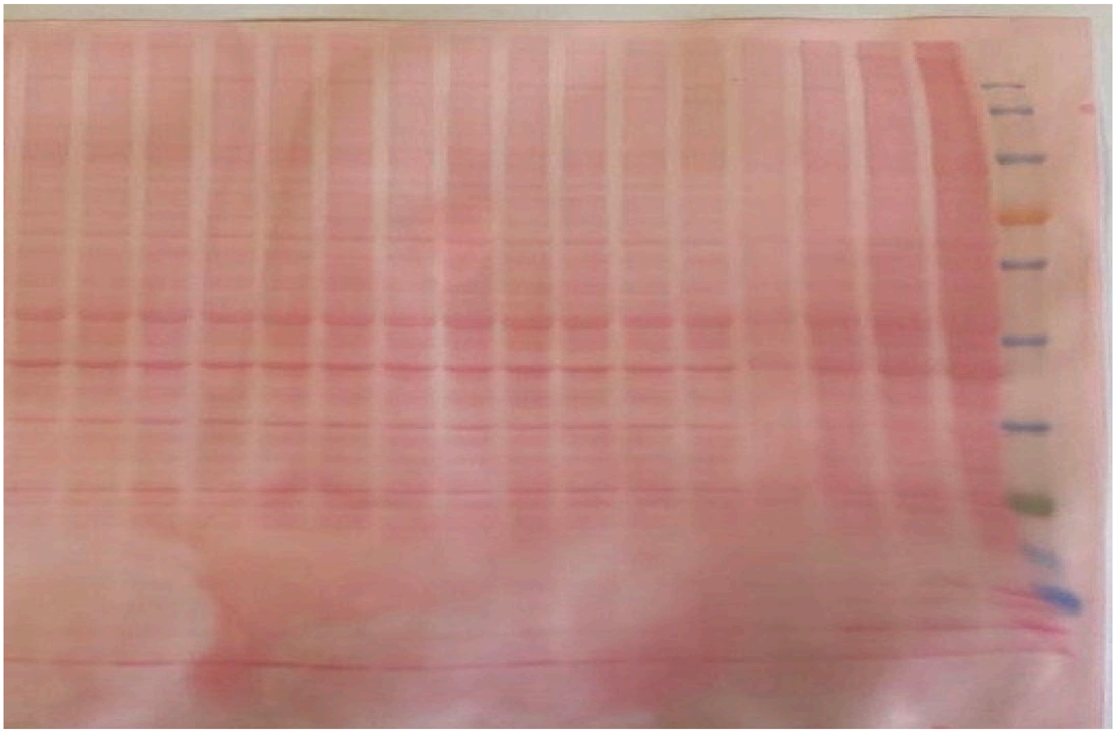

Supplementary Figure S9. Ponceau S stained image of nitrocellulose membrane (BLA) further used for SNAP 25 immunoblotting. Last lane MW standards. Four lanes before last - internals standards (15, 30,45 and 60 mkg of total protein).

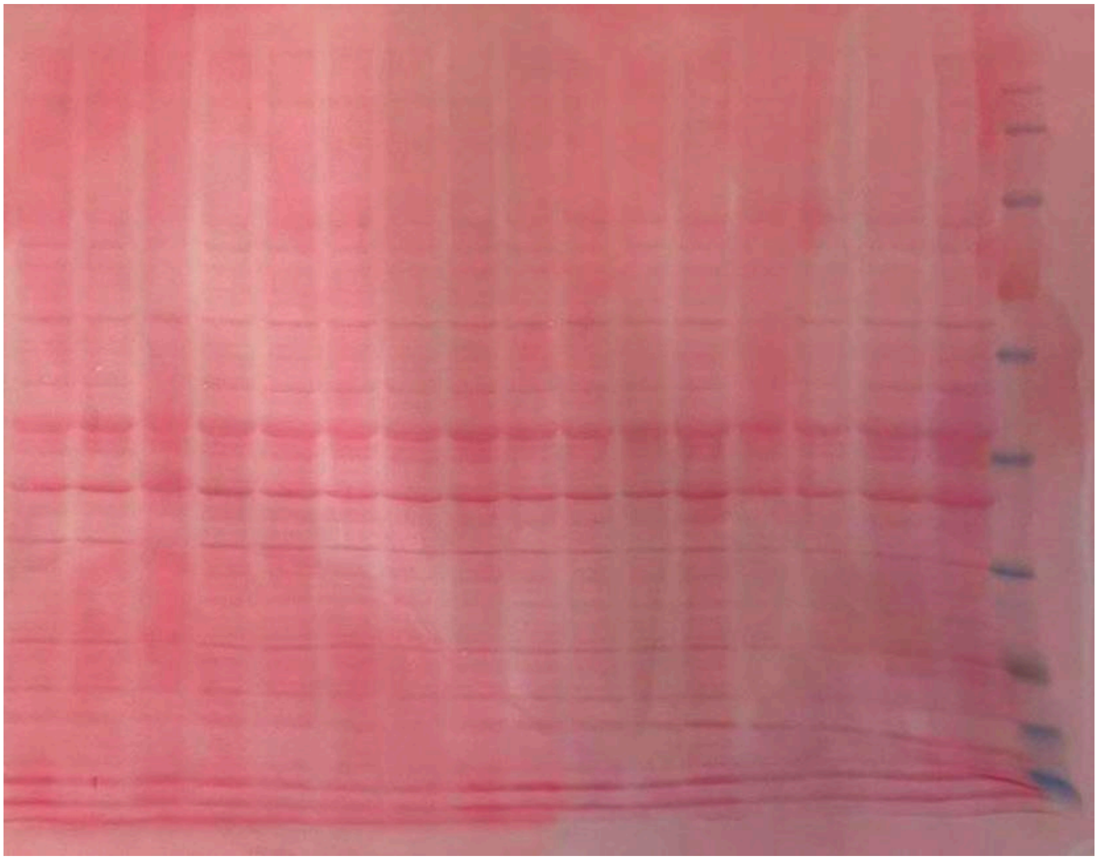

Supplementary Figure S10. Ponceau S stained image of nitrocellulose membrane (Hippocampus) further used for **AIM2 and NLRP** immunoblotting (the membrane is cut into tow parts then). Last lane MW standards. Four lanes before last - internals standards (15, 30,45 and 60 mkg of total protein).

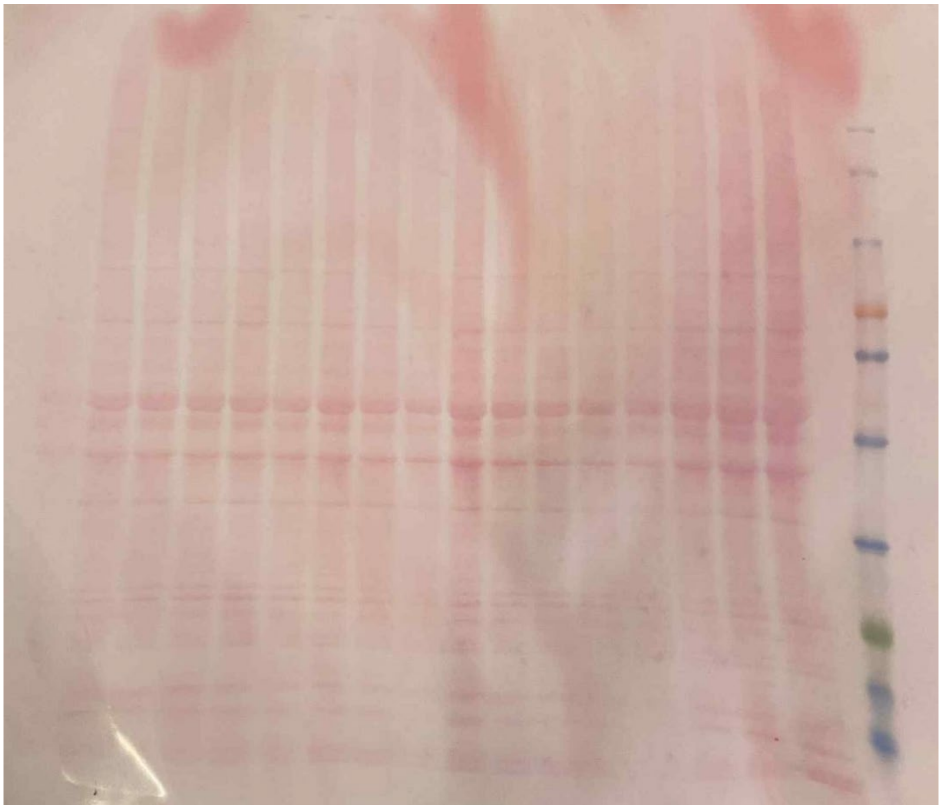

Supplementary Figure S11. Ponceau S stained image of nitrocellulose membrane (BLA) further used for AIM2 and NLRP immunoblotting (the membrane is cut into tow parts then). Last lane MW standards. Four lanes before last - internals standards (15, 30,45 and 60 mkg of total protein

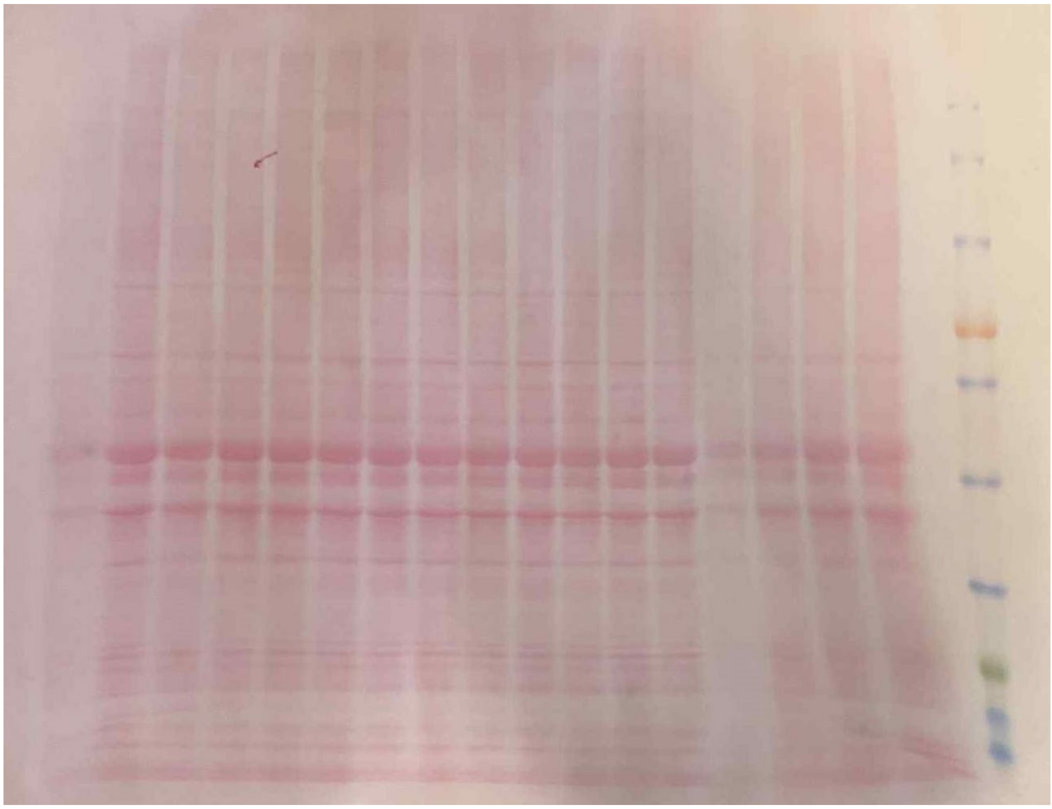

Supplementary Figure S12. Ponceau S stained image of nitrocellulose membrane (CNIC) further used for AIM2 and NLRP immunoblotting (the membrane is cut into tow parts then). Last lane MW standards. Four lanes before last - internals standards (15, 30,45 and 60 mkg of total protein)

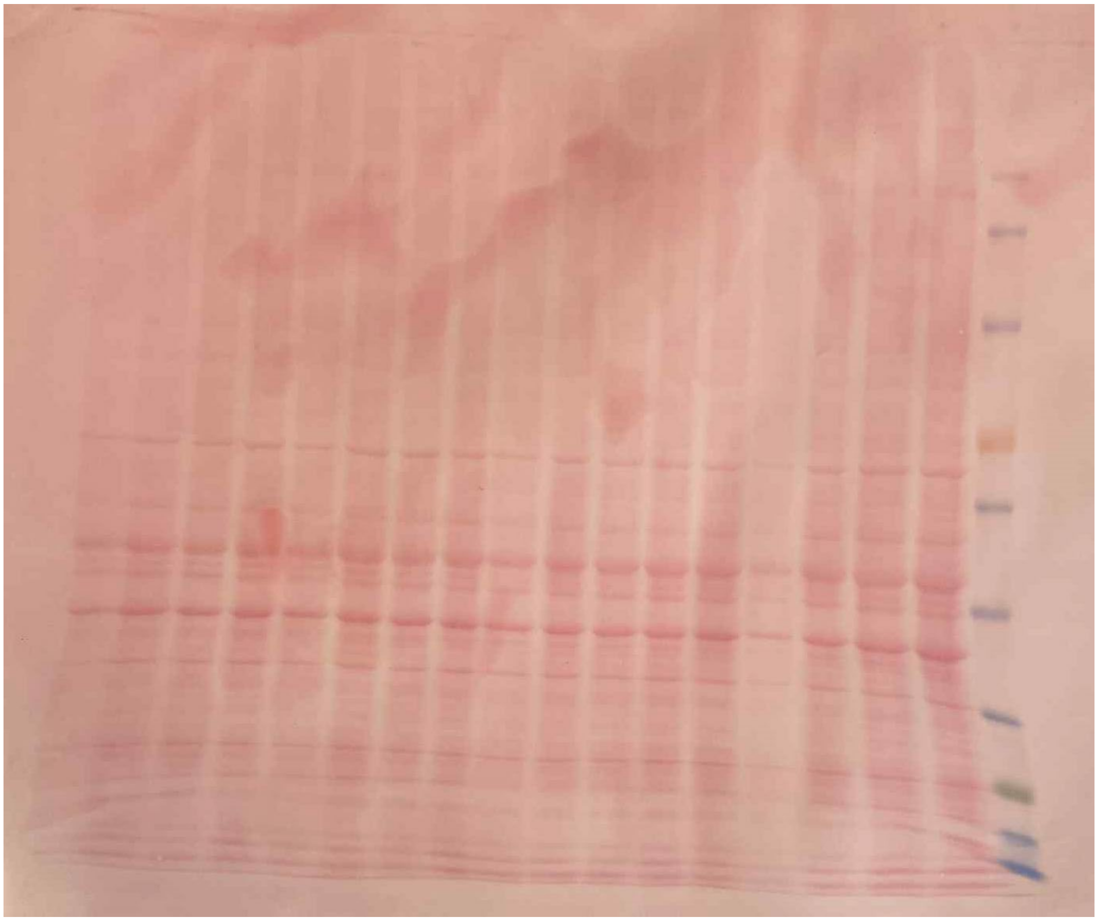

Supplementary Figure S13. Ponceau S stained image of nitrocellulose membrane (Hippocampus) further used for MDA-Protein adducts immunoblotting. Last lane MW standards. Four lanes before last - internals standards (15, 30,45 and 60 mkg of total protein)

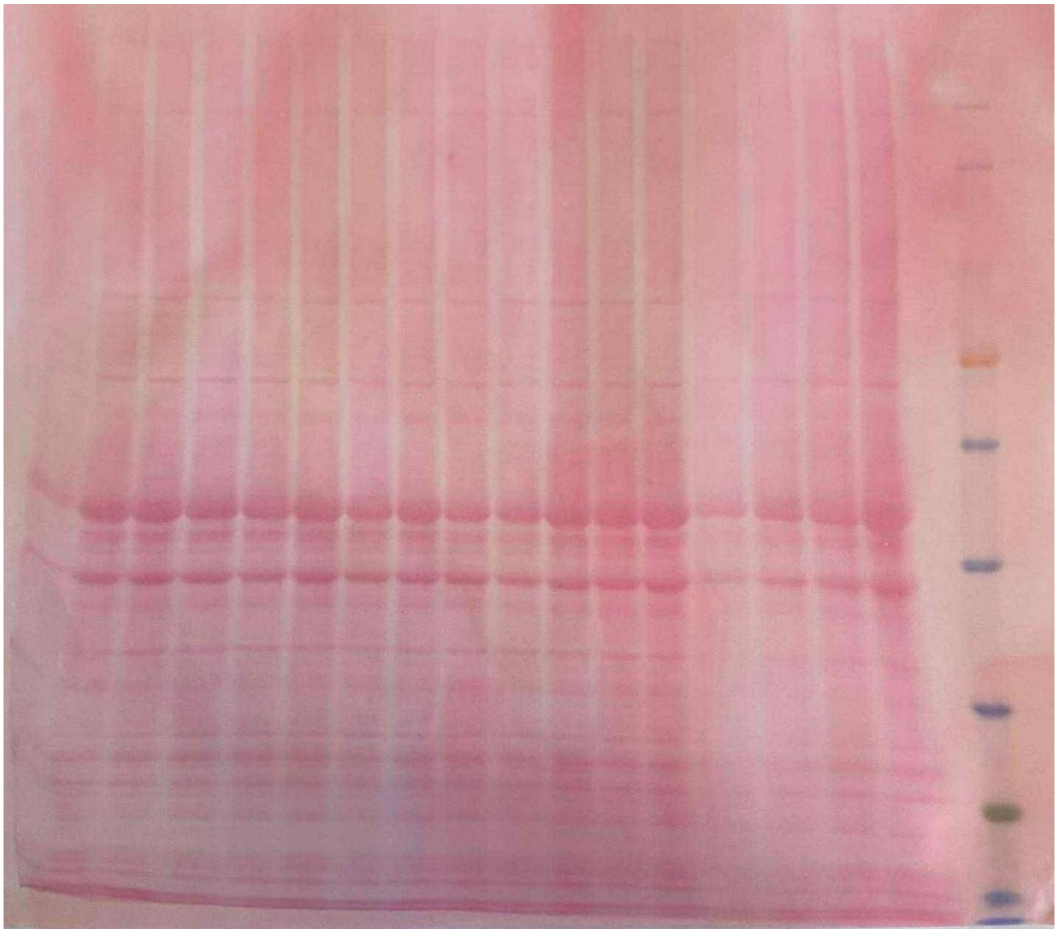

Supplementary Figure S14. Ponceau S stained image of nitrocellulose membrane (BLA) further used for MDA-Protein adducts immunoblotting**.** Last lane MW standards. Four lanes before last - internals standards (15, 30,45 and 60 mkg of total protein).

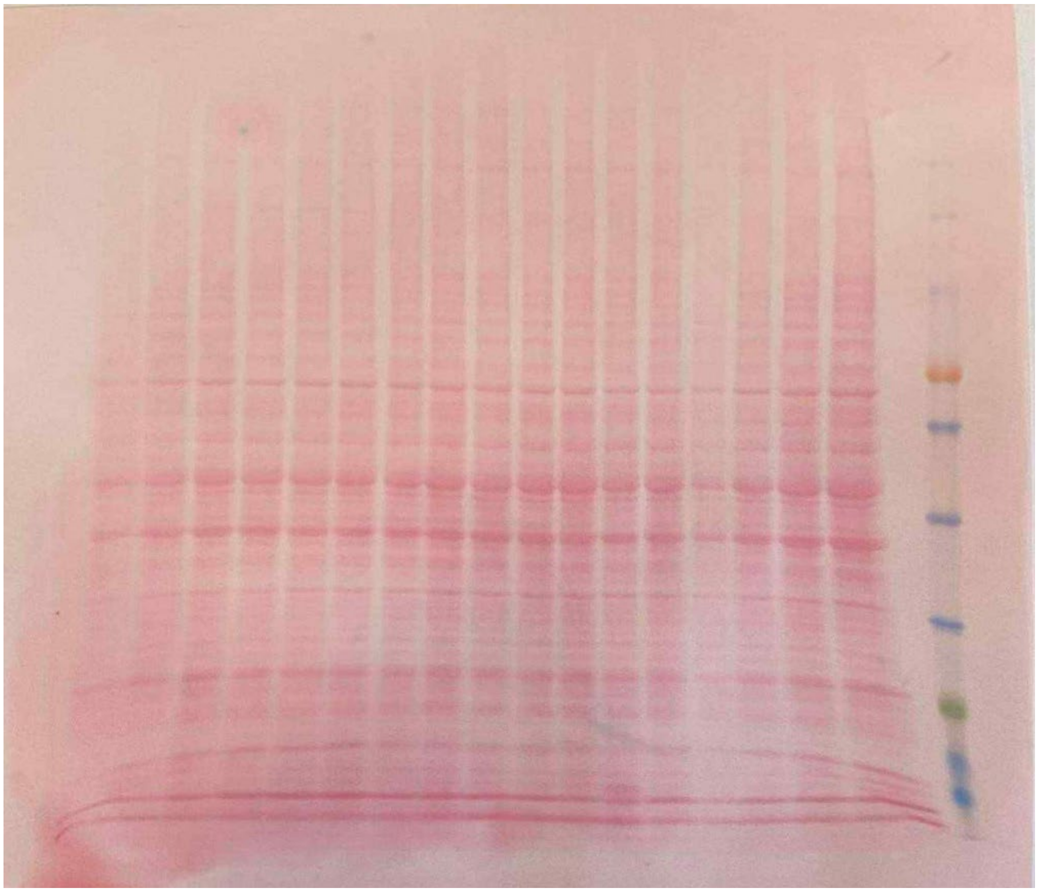

Supplementary Figure S15. Ponceau S stained image of nitrocellulose membrane (CNIC) further used for MDA-Protein adducts immunoblotting**.** Last lane MW standards. Four lanes before last - internals standards (15, 30,45 and 60 mkg of total protein).

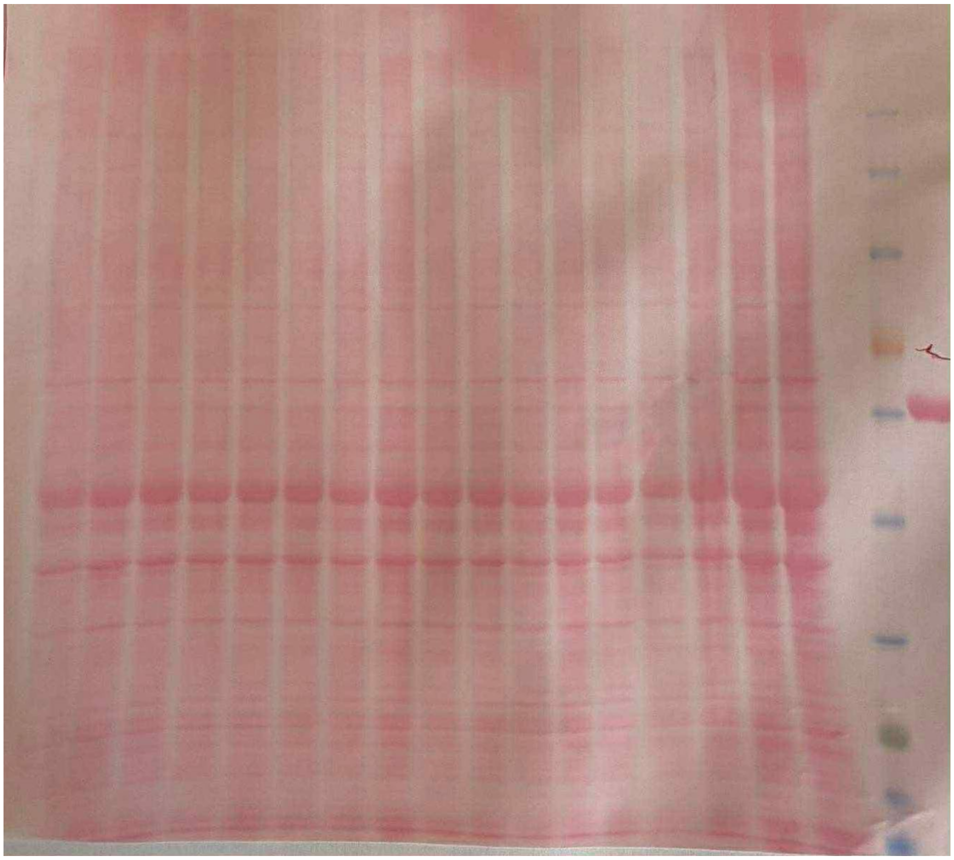

Supplementary Figure S16. Ponceau S stained image of nitrocellulose membrane (Hippocampus) further used for mitochondrial OXPHOS system and ATP synthase F1 subunit alpha (ATP5A) immunoblotting**.** Last lane MW standards. Four lanes before last - internals standards (15, 30,45 and 60 mkg of total protein)

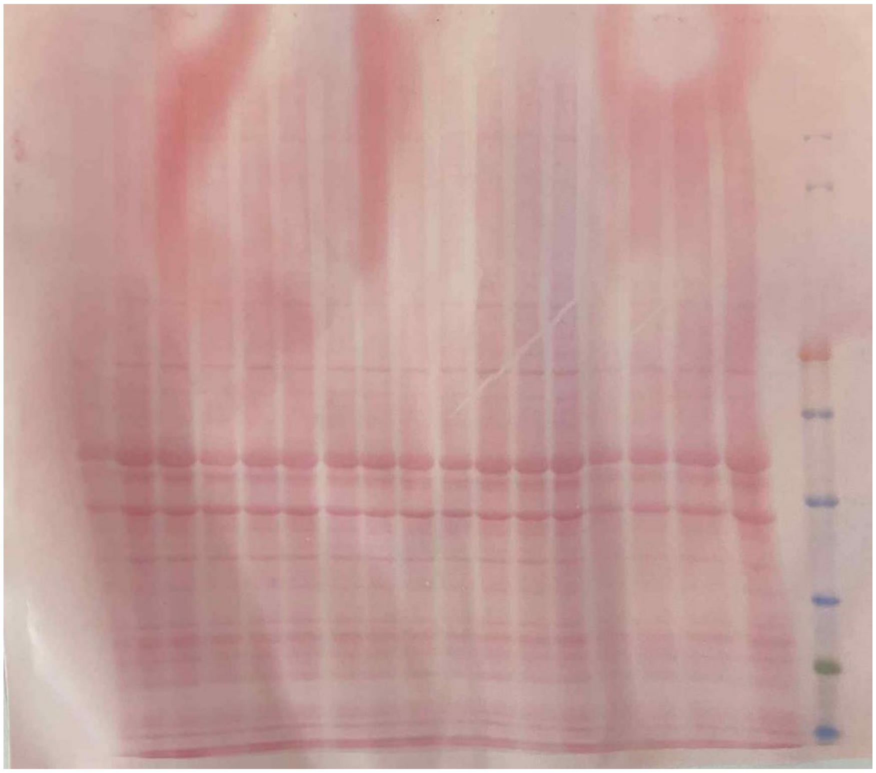

Supplementary Figure S17. Ponceau S stained image of nitrocellulose membrane (BLA) further used for mitochondrial OXPHOS system and ATP synthase F1 subunit alpha (ATP5A) immunoblotting**.** Last lane MW standards. Four lanes before last - internals standards (15, 30,45 and 60 mkg of total protein)

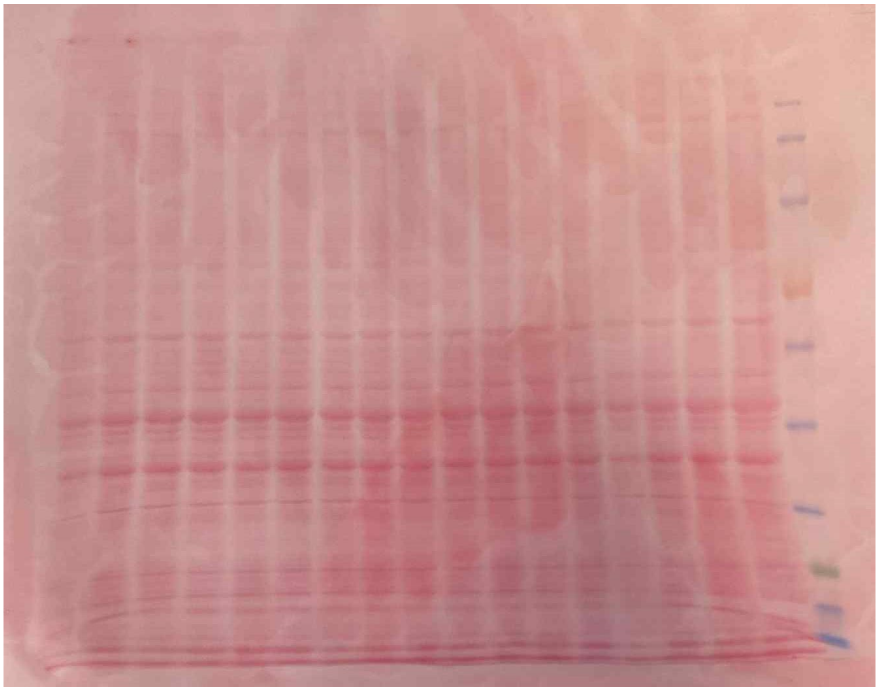

Supplementary Figure S18. Ponceau S stained image of nitrocellulose membrane (CNIC) further used for mitochondrial OXPHOS system and ATP synthase F1 subunit alpha (ATP5A) immunoblotting**.** Last lane MW standards. Four lanes before last - internals standards (15, 30,45 and 60 mkg of total protein)

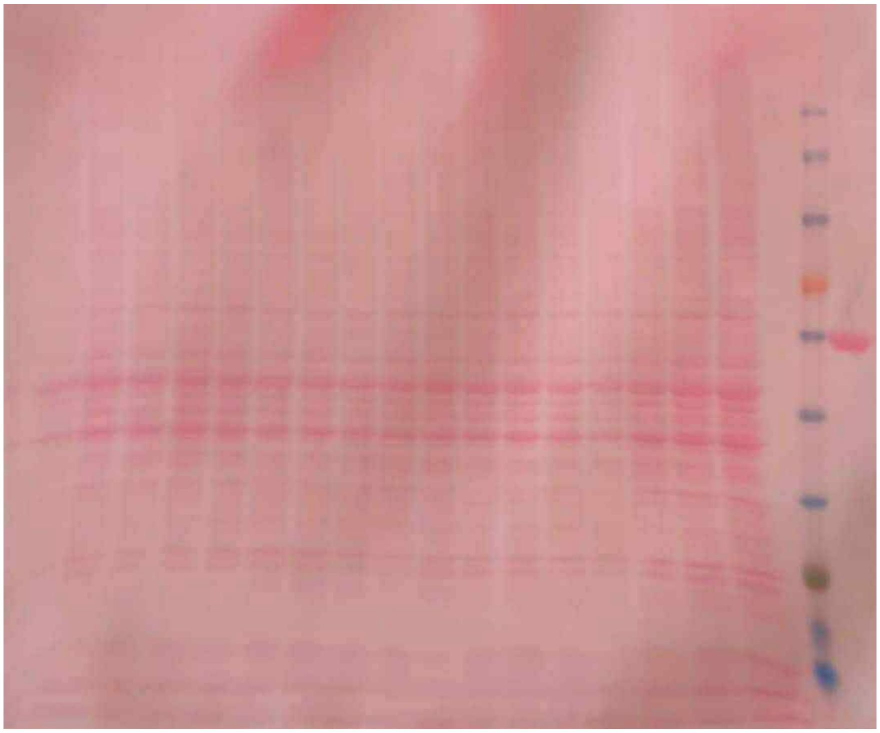

Supplement: Supplementary file 1 — Supporting Information 1 Figures S1–S18: Ponceau S stained image of all nitrocellulose membranes. [file BMRI-2026-2680036-s001.pdf]
