# Supplementary material for: Chronic Loud Noise—Biochemical and Ultrastructural Alterations in Auditory and Limbic Regions of the Rat Brain
Source: Biomed Res Int. 2026 Apr 24;2026:2680036. doi: 10.1155/bmri/2680036 (PMC13108246; doi:10.1155/bmri/2680036)

Supplementary Figure S19.

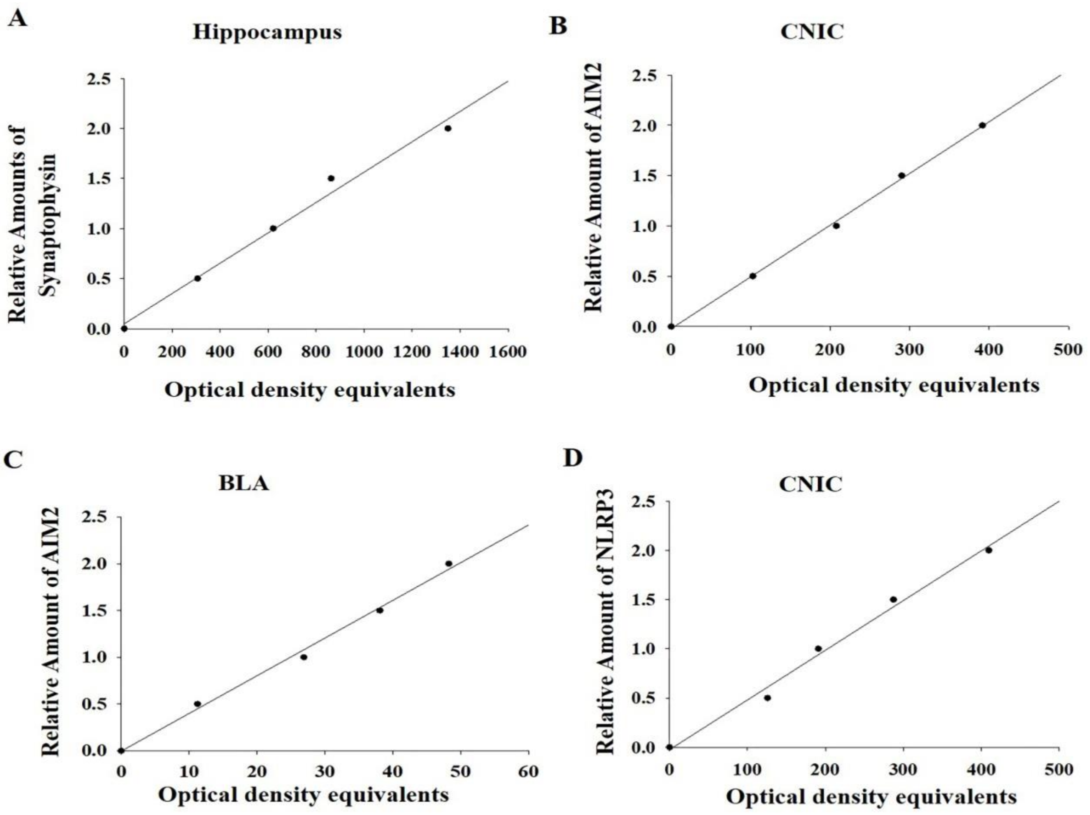

Supplementary Figure S20.

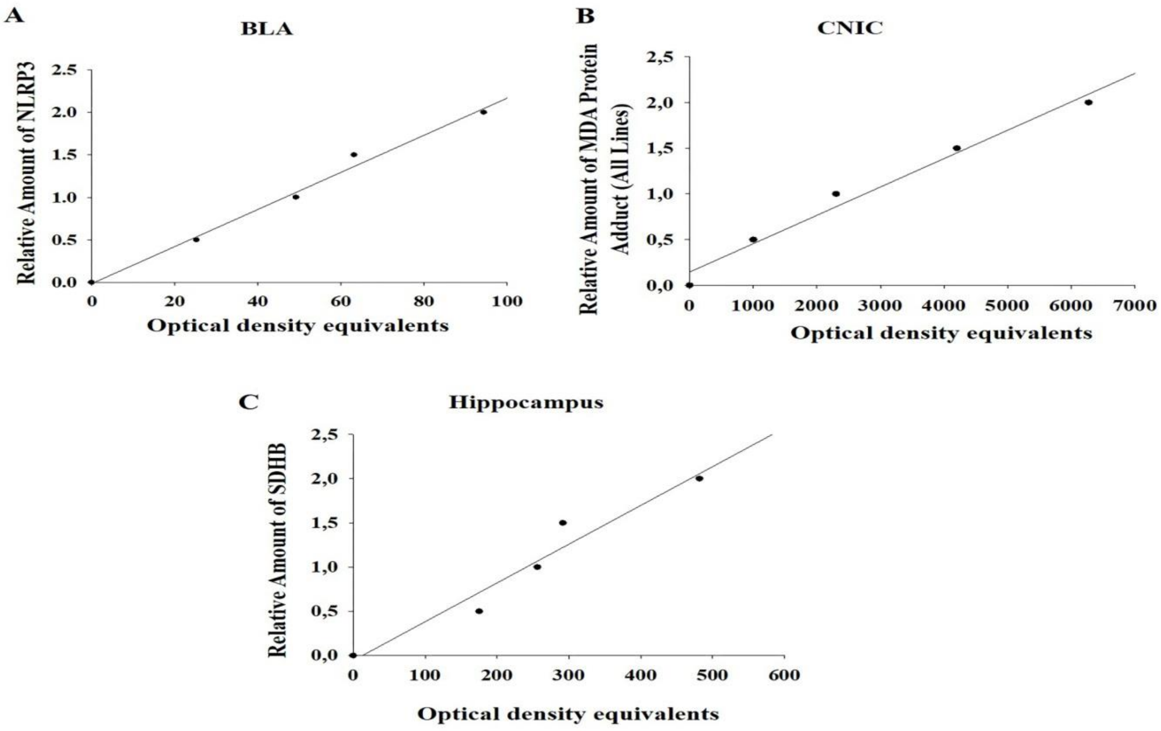

Supplementary Figure S21.

Synaptophysin-CNIC

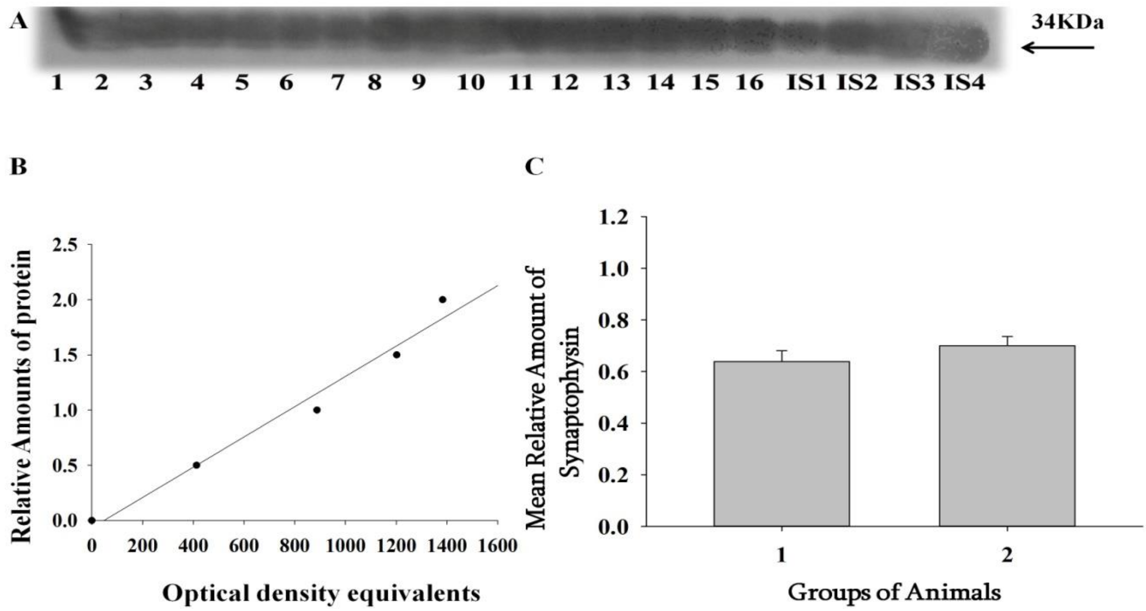

Supplementary Figure S22.

Synaptophysin- BLA

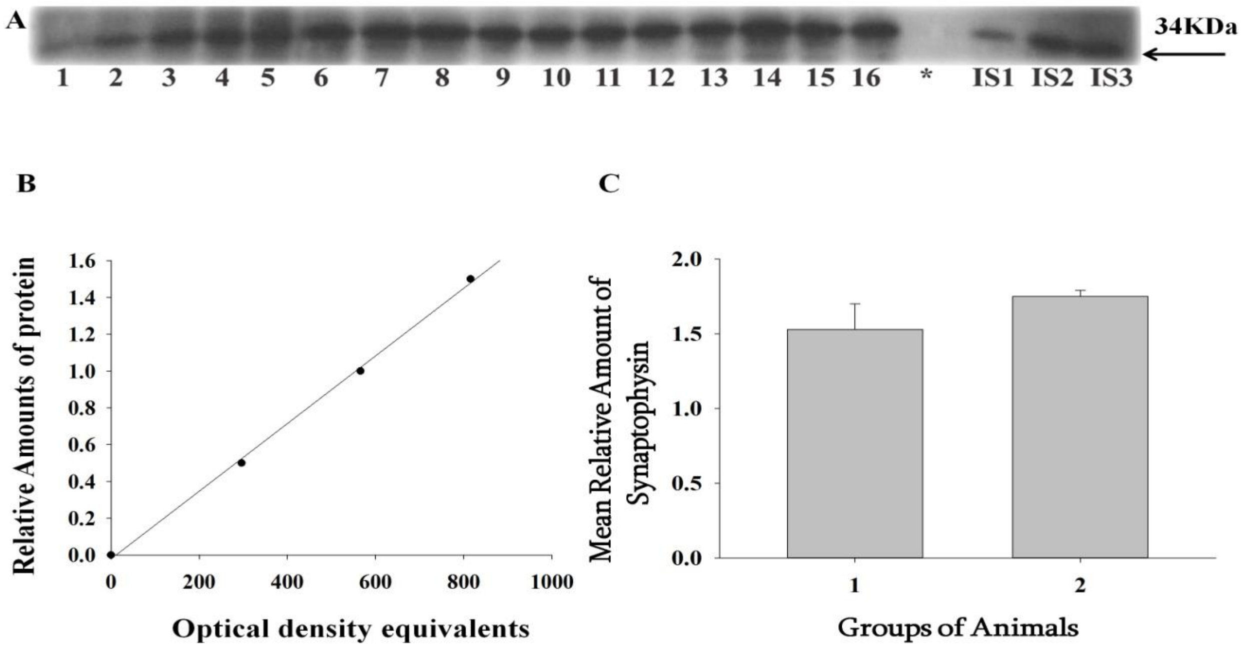

Supplementary Figure S23.

SNAP 25- Hippocampus

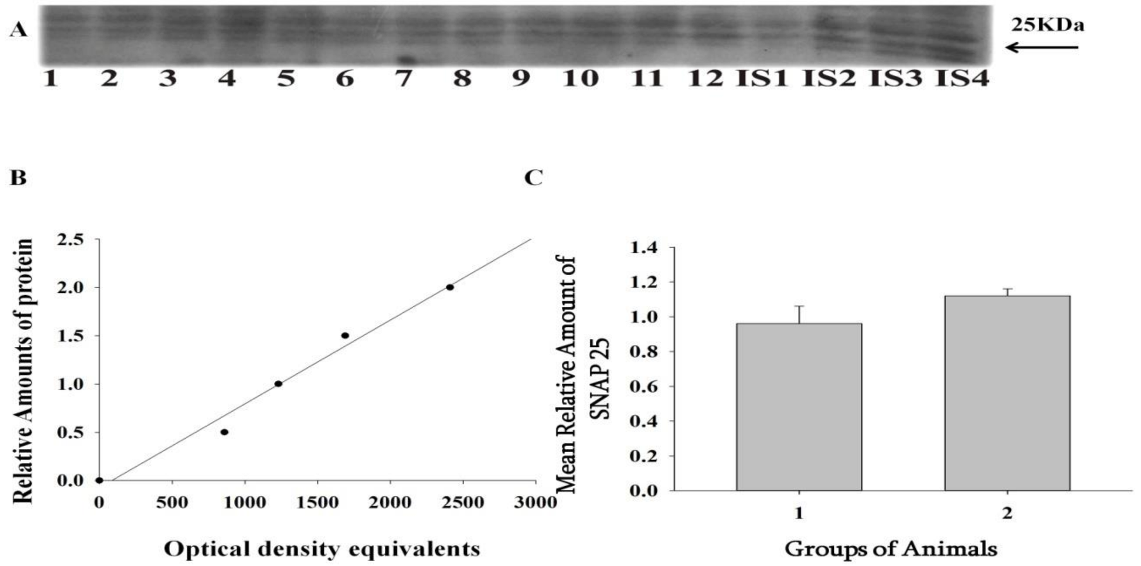

Supplementary Figure S24.

SNAP25-BLA

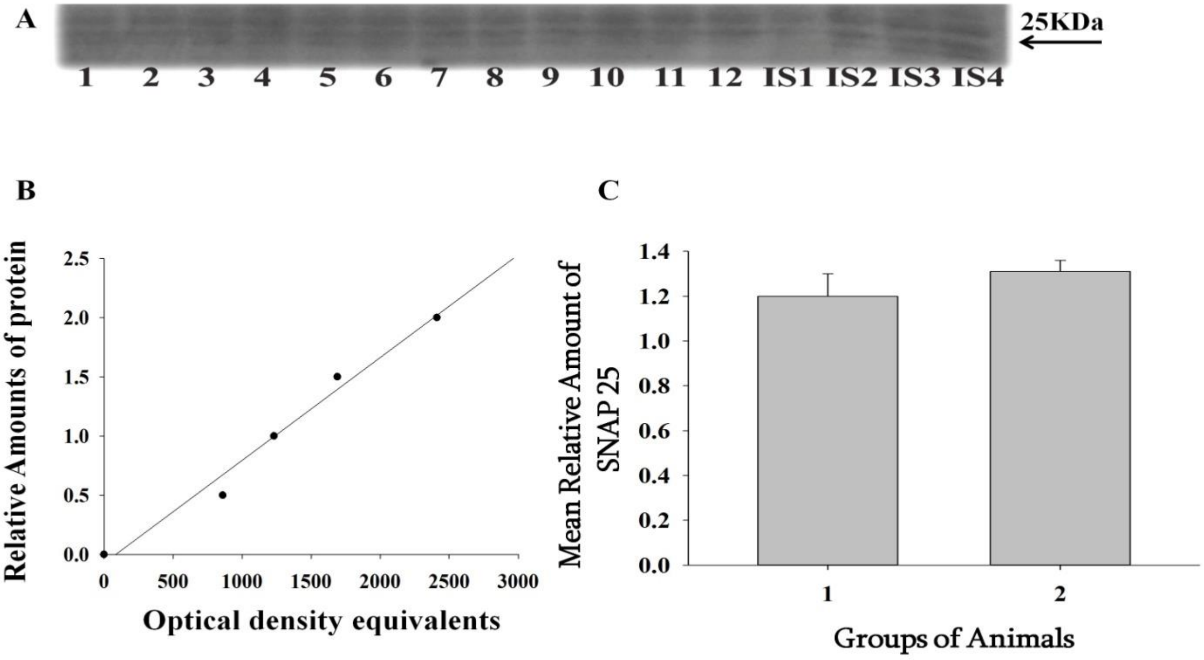

Supplementary Figure S25.

SNAP 25- CNIC

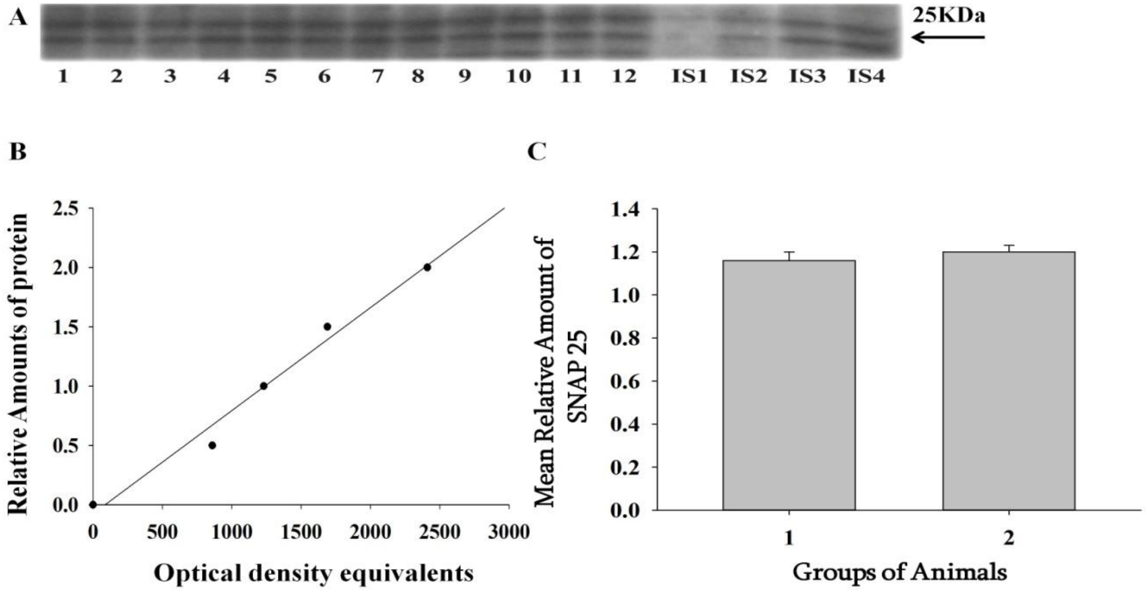

Supplementary Figure S26.

Syntaxin 1 a-hippocampus

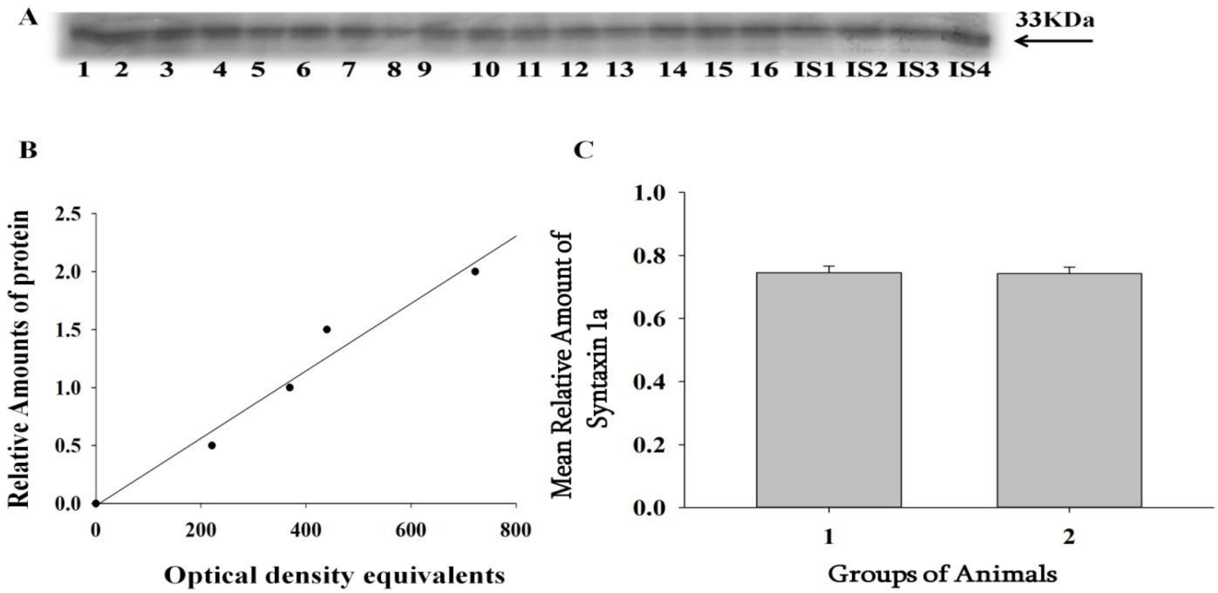

Supplementary Figure S27.

Syntaxin 1a-BLA

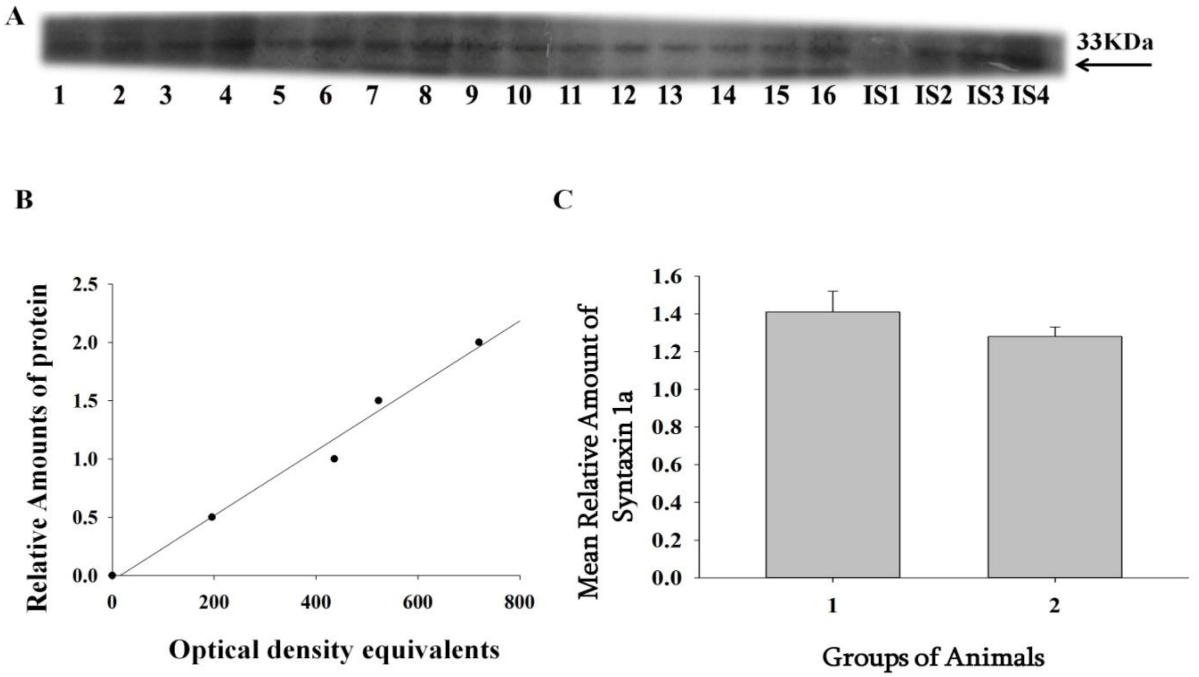

Supplementary Figure S28.

Syntaxin 1 a-CNIC

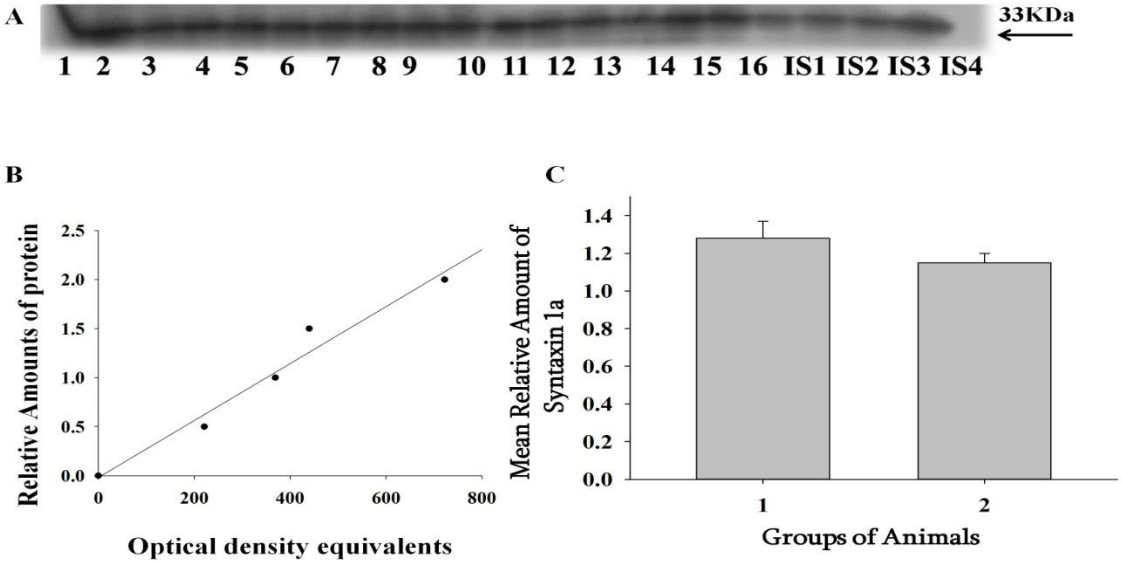

Supplementary Figure S29.

AIM2 - Hippocampus

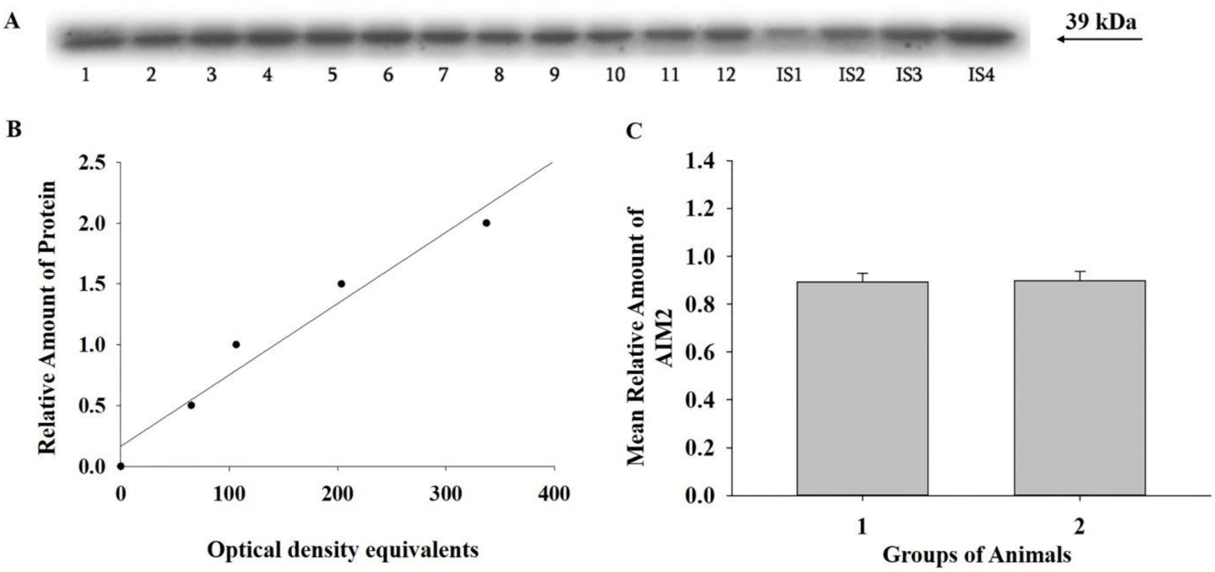

Supplementary Figure S30.

NLRP3 - Hippocampus

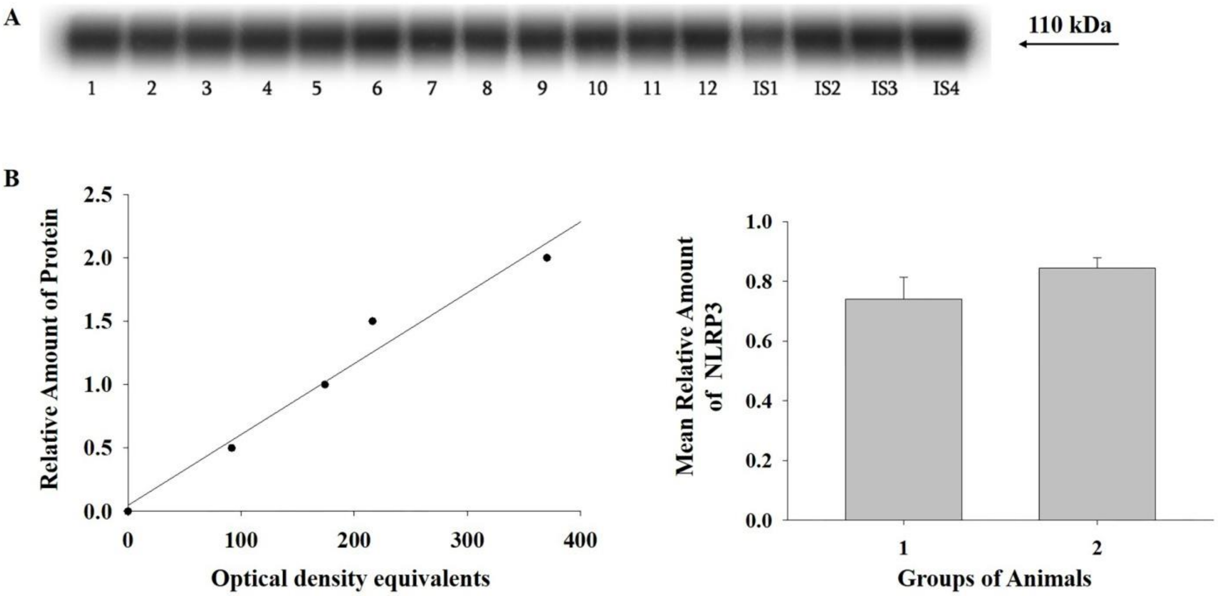

Supplementary Figure S31.

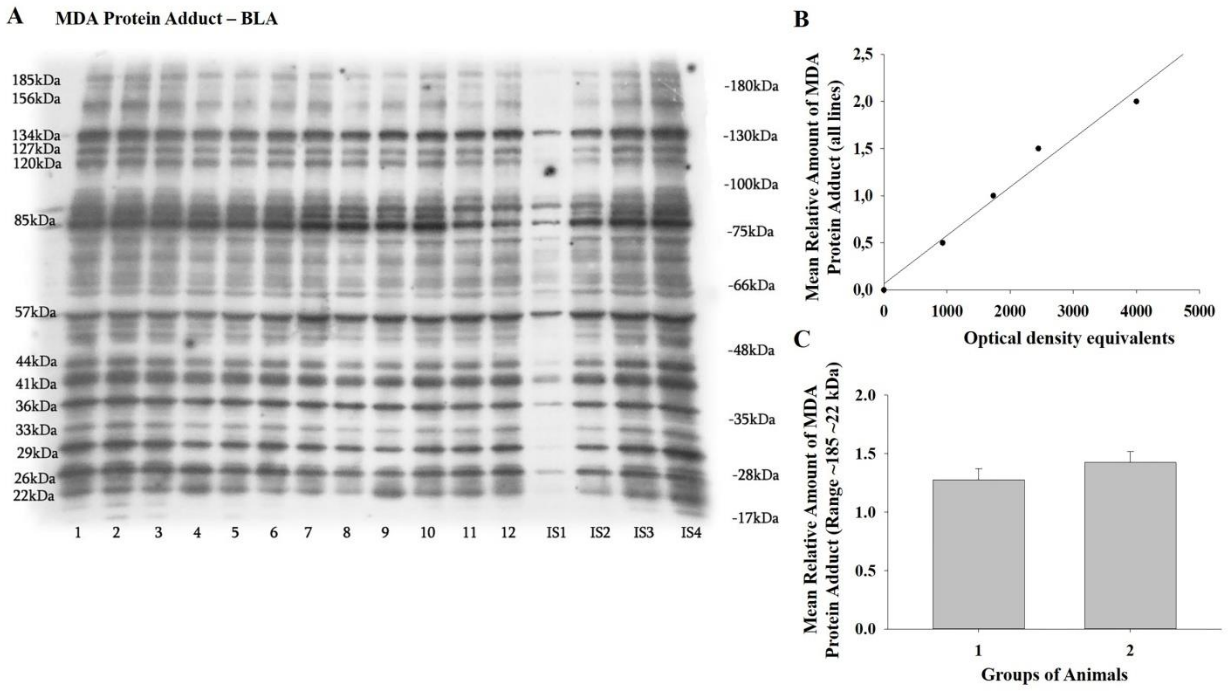

Supplementary Figure S32.

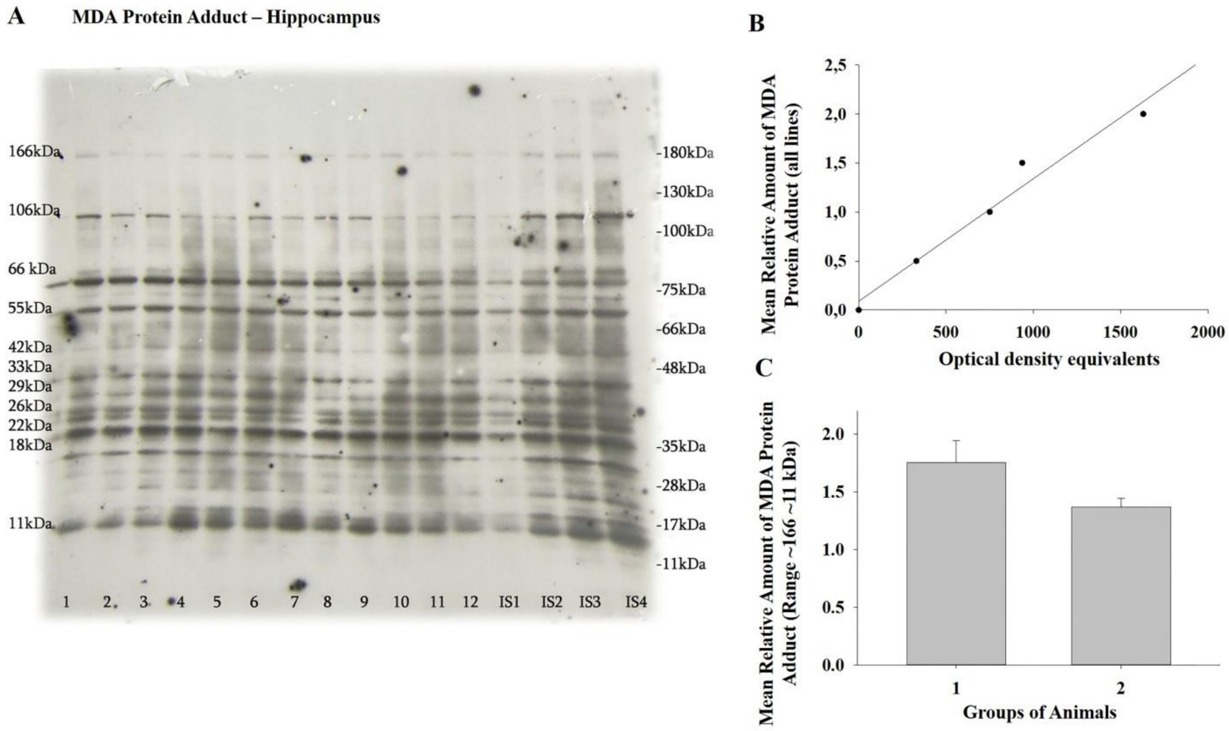

Supplementary Figure S33.

OXPHOS - CNIC

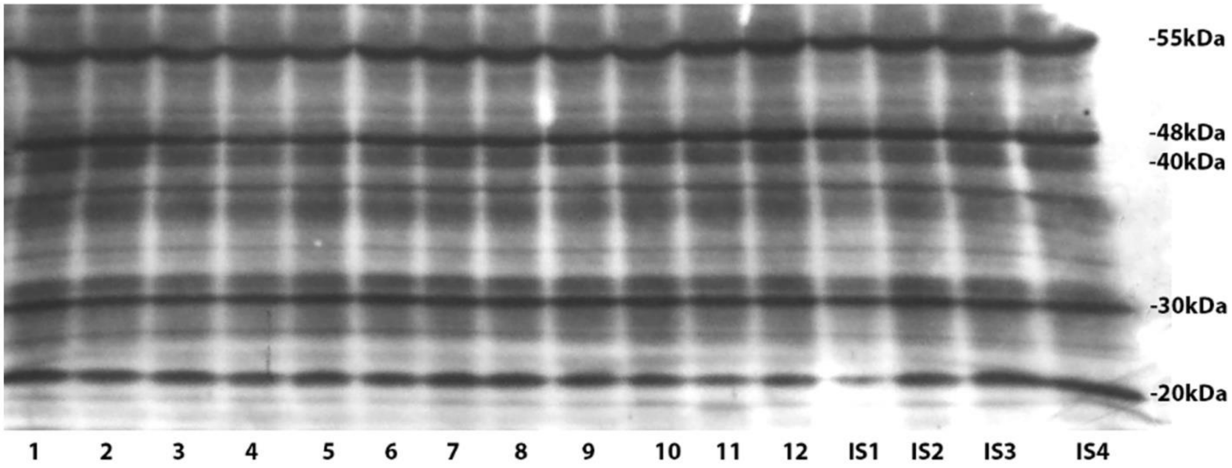

Supplementary Figure S34.

OXPHOS - BLA

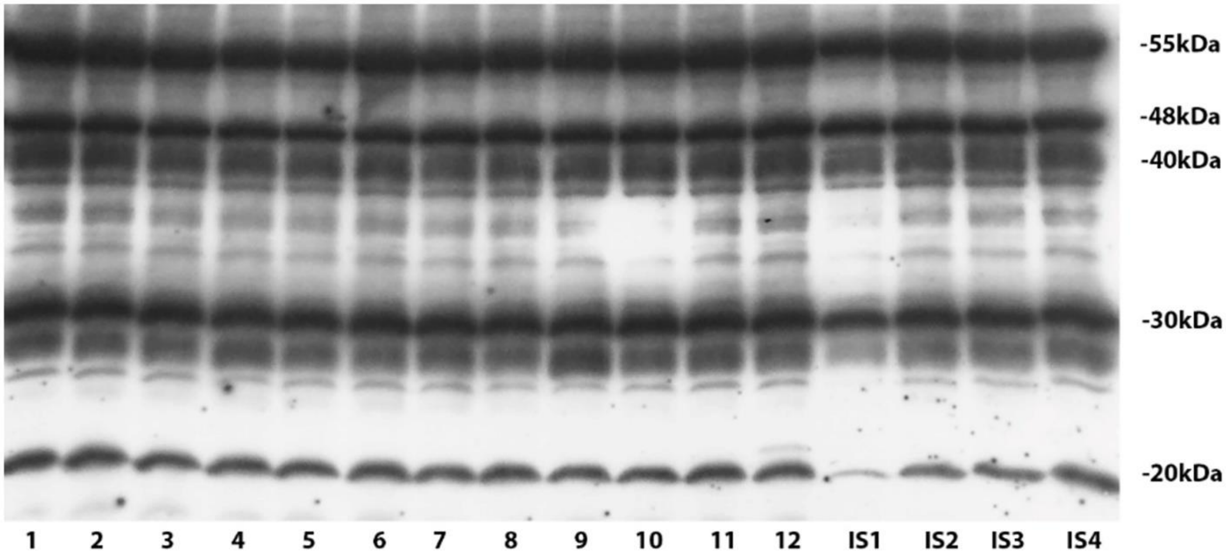

Supplement: Supplementary file 2 — Supporting Information 2 Figure S19: Calibration plots of optical density of internal standards with the amount of loaded protein for: (A) Synaptophysin, hippocampus; (B) AIM2 and CNIC; (C) AIM2 and BLA; and (D) NLRP3 and CNIC. Figure S20: Calibration plots of optical density of internal standards with the amount of loaded protein for: (A) NLRP3 and BLA; (B) MDA‐protein adducts and CNIC; and (C) SDHB and CA1. Figure S21: (A) Western blot image, (B) calibration plot, and (C) mean relative amounts ± sem of synaptophysin in BLA in control and noise‐exposed groups of male Wistar rats. Figure S22: (A) Western blot image, (B) calibration plot, and (C) mean relative amounts ± sem of Synaptophysin in CNIC in control and noise‐exposed groups of male Wistar rats. Figure S23: (A) Western blot image, (B) calibration plot, and (C) mean relative amounts ± sem of SNAP25 in CA1 in control and noise‐exposed groups of male Wistar rats. Figure S24: (A) Western blot image, (B) calibration plot, and (C) mean relative amounts ± sem of SNAP25 in BLA in control and noise‐exposed groups of male Wistar rats. Figure S25: (A) Western blot image, (B) calibration plot, and (C) mean relative amounts ± sem of SNAP25 in CNIC in control and noise‐exposed groups of male Wistar rats. Figure S26: (A) Western blot image, (B) calibration plot, and (C) mean relative amounts ± sem of syntaxin1a in CA1 in control and noise‐exposed groups of male Wistar rats. Figure S27: (A) Western blot image, (B) calibration plot, and (C) mean relative amounts ± sem of Syntaxin1a in BLA in control and noise‐exposed groups of male Wistar rats. Figure S28: (A) Western blot image, (B) calibration plot, and (C) mean relative amounts ± sem of syntaxin1a in CNIC in control and noise‐exposed groups of male Wistar rats. Figure S29: (A) Western blot image, (B) calibration plot, and (C) mean relative amounts ± sem of AIM2 in CA1 in control and noise‐exposed groups of male Wistar rats. Figure S30: (A) Western blot image, (B) calibrat [file BMRI-2026-2680036-s002.pdf]
