# Supplementary material for: Chronic Loud Noise—Biochemical and Ultrastructural Alterations in Auditory and Limbic Regions of the Rat Brain
Source: Biomed Res Int. 2026 Apr 24;2026:2680036. doi: 10.1155/bmri/2680036 (PMC13108246; doi:10.1155/bmri/2680036)
Supplement: Supplementary file 3 — Supporting Information 3 File S1 [Table S1]: Power calculation for sample size; File S2: Immunoblotting information; File S3 [TableS2]: Mean relative amounts of synaptophysin in CNIC, hippocampus, and BLA; File S4 [Table S3]: Mean relative amounts of SNAP25 in CNIC, hippocampus, and BLA; File S5 [Table S4]: Mean relative amounts of syntaxin in CNIC, hippocampus, and BLA; File S6 [Table S5]: Mean relative amounts of AIM2 and NLRP3 in CNIC, hippocampus, and BLA; File S7 [Table S6]: Mean relative amounts of different MDA‐protein adducts in IC. [file BMRI-2026-2680036-s003.pdf]

Supplementary File-S1 (Supplementary Table- S1)-Power analysis calculation

Power analysis calculation

Standard deviation	Delta (16 samples)	Delta (12 samples)
0.2	0.301329478	0.3591094
0.4	0.602658956	0.7182188
0.6	0.903988433	1.0773282
0.8	1.205317911	1.4364376
1.0	1.506647389	1.795547

Supplementary File-S2-Immunoblotting

Immunoblotting

Antibodies against synaptophysin, SNAP25, syntaxin, AIM2 and NLRP reacted with protein bands of 34, 25, 33, 39 and 110 kDa respectively. A cocktail of antibodies against components of oxidative phosphorylation and ATP synthase F1 subunit alpha immunostained protein bands of 55, 48, 40, 30 and 20 kDa molecular weight corresponding to ATP5A, UQCRC2, MTCO, SDHB and NDUFB8 respectively. Antibodies against MDA reacted with different protein molecules (Figures 1-7, Supplementary Figures S19-S34). Four standards, 15, 30, 45, and 60 μg of homogenate protein corresponding to 0.5, 1.0, 1.5 and 2.0 relative amounts of protein, respectively, were loaded on each gel. For all studied proteins the optical densities of these internal standards were plotted against the amount of protein. For all measurements least-squares regression showed a perfect fit to a straight line (Supplementary Figures S19-S34).

Supplementary File S3 [Supplementary Table-S2]. Mean relative amount Synaptophysin in CNIC, Hippocampus and BLA

**Brain region**	White Noise	CONTROL	P values, T and DF
	Mean relative amount Synaptophysin ±sem		
**CNIC**	0.704<br>±0,038	0,639<br>±0,03	0,208<br>-1,34<br>14
**Hippocampus**	0.863<br>±0,03	0,632<br>±0,03	0,001<br>-4,79<br>14
**BLA**	1.754<br>±0,046	1,539<br>±0,17	0,093<br>-1,81<br>14

Supplementary File S4 [Supplementary Table S3]. Mean relative amount SNAP25 **in** CNIC, Hippocampus and BLA

**Brain region**	White Noise	CONTROL	P values, T and DF
	Mean relative amount SNAP25 ±sem		
**CNIC**	1.2060<br>±0,038	1,1639<br>±0,04	0,479<br>-0,734

			12
**Hippocampus**	1.123<br>±0,048	0,932<br>±0,10	0,121<br>-1,67<br>12
**BLA**	1.311<br>±0,058	1,159<br>±0,11	0,246<br>-1,22<br>12

Supplementary File S5 [Supplementary Table S4]. Mean relative amount Syntaxin **in** CNIC, Hippocampus and BLA

**Brain region**	White Noise	CONTROL	P values, T and DF
	Mean relative amount Syntaxin ±sem		
**CNIC**	1.15<br>±0,09	1.289<br>±0,09	0,251<br>1.20<br>14
**Hippocampus**	0.732<br>±0,029	0,746<br>±0,029	0.742<br>0.34<br>14
**BLA**	1.282<br>±0,085	1,414<br>±0,11	0,361<br>0,94<br>14

Supplementary File S6 [Supplementary Table S5]. Mean relative amounts of AIM2 and NLRP3 in CNIC, Hippocampus and BLA

**Brain region**	White Noise	CONTROL	P values, T and DF	White Noise	CONTROL	P values, T and DF
	Mean relative amount **AIM2 -** ±sem			Mean relative amount **NLRP3 -** ±sem		
**CNIC**	0.9833± 0.037	0.819± 0.046	0.020 -2.77 10	0.8325± 0.0083	0.6839± 0.031	0.001 -4.63 10
**Hippocampus**	0,8984± 0,039	0,8926± 0,035	0.914 -0.11 10	0.8441± 0.034	0.741± 0.072	0.223 -1.30 10
**BLA**	0.4278± 0.023	0.6510± 0.030	0.000 5.86 10	0.701± 0.044	0.8448± 0.037	0.033 2.48 10

Supplementary File S7 [Supplementary Table S6]. Mean relative amounts of different MDA-Protein adducts in IC

MDA-protein adducts by molecular weight	**White Noise**	**CONTROL**	**Ratio WN/CONT, P values, T and DF**
	**IC**		
Mean relative amount **MDA-**	1,095	0,526	2,0813

**Protein adducts** 162 kDa ±sem	±0,05	±0,081	0,000<br>-5,99<br>10
Mean relative amount **MDA-Protein adducts** 89 kDa ±sem	1,474<br>±0,062	0,851<br>±0,10	1,7318<br>0,000<br>-5,17<br>10
Mean relative amount **MDA-Protein adducts** 74 kDa ±sem	0,958<br>±0,047	1,054<br>±0,068	0,9084<br>0,269<br>1,17<br>10
Mean relative amount **MDA-Protein adducts** 57 kDa ±sem	1,329<br>±0,073	0,886<br>±0,055	1,5013<br>0,001<br>-4,83<br>10
Mean relative amount **MDA-Protein adducts** 50 kDa ±sem	1,653<br>±0,069	1,308<br>±0,090	1,2634<br>0,012<br>-3,05<br>10
Mean relative amount **MDA-Protein adducts** 44 kDa ±sem	2,088<br>±0,080	1,446<br>±0,11	1,4441<br>0,001<br>-4,64<br>10
Mean relative amount **MDA-Protein adducts** 36 kDa ±sem	1,686<br>±0,073	1,583<br>±0,11	1,0649<br>0,445<br>-0,79<br>10
Mean relative amount **MDA-Protein adducts** 32 kDa ±sem	1,524<br>±0,091	1,366<br>±0,080	1,1157<br>0,222<br>-1,30<br>10

Mean relative amount **MDA-Protein adducts** 26kDa ±sem	1,084<br>±0,041	0,869<br>±0,066	1,2469<br>0,021<br>-2,75<br>10
Mean relative amount **MDA-Protein adducts** 24kDa ±sem	1,669<br>±0,026	1,429<br>±0,051	1,1684<br>0,002<br>-4,19<br>10
Mean relative amount **MDA-Protein adducts** 21kDa ±sem	1,693<br>±0,065	1,163<br>±0,15	1,4551<br>0,008<br>-3,33<br>10
Mean relative amount **MDA-Protein adducts** (all kDa lines) ±sem	1,437<br>0,064	0,998<br>0,088	1,4404<br>0,002<br>-4,03<br>10
